# Targeted inhibition of MDSC-derived exosomal miR-155-5p restrains epithelial-mesenchymal transition in hormone receptor-positive breast cancer by regulating SIRT1

**DOI:** 10.1016/j.mtbio.2025.102492

**Published:** 2025-10-31

**Authors:** Guidong Chen, Silei Wang, Fanchen Wang, Chenju Yang, Rui Zhang, Pengpeng Liu, Junya Ning, Shuyu Wang, Feihe Ma, Linlin Xu, Linqi Shi, Jinpu Yu

**Affiliations:** a Cancer Molecular Diagnostics Core, Tianjin Medical University Cancer Institute and Hospital, National Clinical Research Center for Cancer, State Key Laboratory of Druggability Evaluation and Systematic Translational Medicine, Tianjin's Clinical Research Center for Cancer, Tianjin, 300060, China; b Key Laboratory of Breast Cancer Prevention and Therapy, Tianjin Medical University, Ministry of Education, Tianjin, 300060, China; c Key Laboratory of Cancer Immunology and Biotherapy, Tianjin, 300060, China; d Key Laboratory of Functional Polymer Materials of Ministry of Education, State Key Laboratory of Medicinal Chemical Biology, Institute of Polymer Chemistry, College of Chemistry, Nankai University, Tianjin, 300071, China

**Keywords:** Hormone receptor-positive breast cancer, Myeloid-derived suppressor cell, MiR-155-5p, SIRT1, Polymeric micelle

## Abstract

Breast cancer is one the most common cancers in women, with 70 % of cases being hormone receptor-positive (HR+), and recurrence and distant metastasis are the leading causes of mortality. Myeloid-derived suppressor cells (MDSCs) are one of the main immunosuppressive cell subsets within the tumor microenvironment (TME) and we previously demonstrated that PIK3CA mutation could induce immune evasion by recruiting MDSCs in breast cancer. However, the direct role of MDSCs in regulating breast cancer cells to promote tumor progression remains unclear. In this study, we found that MDSCs co-incubation enhanced the migration and invasion of HR + breast cancer cells. Additionally, exosomal miR-155-5p from MDSCs was shown to downregulate SIRT1 expression in breast cancer cells, thereby promoting epithelial-mesenchymal transition. To address this, we developed a TME-responsive polymeric micelle co-delivering alpelisib (a pan-PI3K inhibitor) and cobomarsen (a miR-155-5p inhibitor) for targeted therapy. The poly(β-amino ester) backbone encapsulated hydrophobic alpelisib for acid-triggered release, while hydrophobized cobomarsen was integrated into the micellar core to improve stability and intracellular delivery. This dual-targeting strategy effectively suppressed PIK3CA-mutated tumor growth and epithelial-mesenchymal transition. These findings reveal the role of MDSC-driven metastatic potential *via* the exosomal miR-155-5p/SIRT1 axis in HR + breast cancer and present a novel nanotherapeutic approach for precision intervention.

## Graphical abstract

A tumor microenvironment-responsive polymeric micelle co-delivering alpelisib and cobomarsen suppresses PIK3CA-mutated HR + breast cancer progression by simultaneously blocking PI3K signaling and MDSC-derived exosomal miR-155-5p/SIRT1 axis-mediated epithelial-mesenchymal transition, offering a promising nanotherapeutic intervention for HR + breast cancer.

**Figure:**
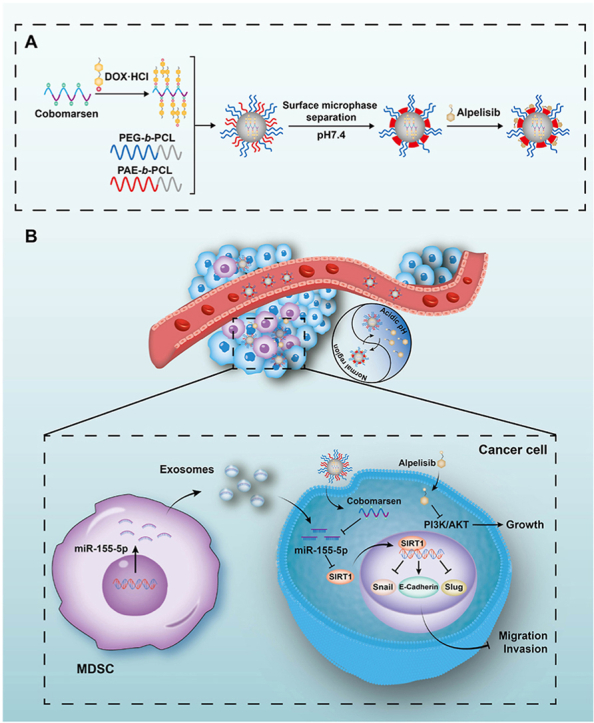

## 1 Introduction

Breast cancer is one of the most prevalent cancers in women, with its incidence steadily rising each year [1]. Although overall mortality remains stable, it continues to pose a significant threat to women's health [2]. The hormone receptor-positive (HR+) subtype accounts for the majority of breast cancer cases, comprising up to 70 %, and typically responds well to endocrine therapy with a favorable prognosis [3,4]. However, approximately 50 % of patients develop resistance to treatment, leading to tumor recurrence and metastatic progression [5]. There is growing evidence that the process of epithelial-mesenchymal transition (EMT), which is an initial step in tumor metastasis, provides cells with the enhanced plasticity necessary for invasion and dissemination to distant sites [6,7]. Moreover, EMT confers properties similar to cancer stem cells upon tumor cells, resulting in increased therapy resistance and a greater propensity for disease recurrence after treatment [8]. Therefore, identifying effective targets for treating HR + metastatic breast cancer has become both critical and urgent.

Oncogenic PIK3CA mutations occur in approximately 30 % of human breast cancers, with a higher incidence in HR + cases [[9], [10], [11]]. These mutations result in abnormal enzyme activity, causing persistent activation of downstream signaling pathways and uncontrolled tumor cell proliferation. Moreover, PIK3CA mutations reprogram the tumor microenvironment (TME): mutant tumor cells promote abnormal blood vessel formation and establish an immunosuppressive niche through the secretion of pro-inflammatory cytokines [12]. Additionally, these mutations activate tumor-associated fibroblasts (CAFs) and skew macrophages toward the M2 phenotype (TAMs), which collectively suppress T cell infiltration and function, rendering PIK3CA-targeted monotherapy ineffective clinically [13].

Our prior study demonstrated that PIK3CA mutations facilitate immune evasion in breast cancer by recruiting myeloid-derived suppressor cells (MDSCs) through activation of the 5-lipoxygenase (5-LOX)-dependent arachidonic acid pathway [14]. Tumor progression and metastasis are complex processes driven by both intrinsic tumor cell malignancy and dynamic remodeling of the TME [15,16]. Concurrently, hypoxia and metabolic stress within the TME activate HIF-1α/NF-κB signaling, which enhances cancer stem cell properties and increases metastatic cell resistance to therapy as well as self-renewal potential [17]. As a major immunosuppressive cell subset within the TME, MDSCs not only exert strong immunosuppressive activity but also facilitate tumor progression and metastasis through direct interactions and paracrine signaling [[18], [19], [20]]. This bidirectional crosstalk between the TME and metastasis highlights the systemic impact of localized microenvironmental reprogramming and presents novel therapeutic targets to combat metastasis. Consequently, investigating the mechanisms by which MDSCs directly regulate breast cancer cells to promote tumor progression is crucial for developing an effective therapeutic strategy for PIK3CA-mutated HR + breast cancer patients.

Exosomes, with diameters ranging from 30 to 100 nm, are key mediators of intercellular communication [21]. These vesicles carry various bioactive cargos-such as proteins, miRNAs, and DNA fragments-to target cells, initiating significant changes in cellular physiology and function [[22], [23], [24]]. Increasing evidence highlights the critical role of exosomes in facilitating the tumor-promoting effects of MDSCs [19,25,26]. As exosome-targeted nanomedicine presents promising opportunities for cancer therapy, developing nanodrugs that target MDSC-derived exosomes may offer a breakthrough in treating PIK3CA-mutant breast cancer [[27], [28], [29]]. However, the precise role of MDSC-derived exosomes in breast cancer remains inadequately understood.

This study aimed to investigate how MDSCs regulate breast cancer cells. Bioinformatics analysis and functional assays identified miR-155-5p as the key exosomal miRNA involved in promoting HR + breast cancer cell migration and invasion through MDSC interaction. Furthermore, miR-155-5p downregulates SIRT1 expression, thereby advancing breast cancer epithelial-mesenchymal transition. Building on these findings, a TME-responsive polymeric micelle that co-delivers cobomarsen and alpelisib was developed, enhancing circulating stability and tumor-selective intracellular accumulation. This system ultimately achieves synergistic anti-tumor efficacy in PIK3CA-mutated tumors. Collectively, our results indicate that MDSC-derived miR-155-5p drives HR + breast cancer progression by suppressing SIRT1, and our engineered TME-responsive polymeric micelle system effectively addresses key pharmacological barriers to synergistic cancer treatment.

## 2 Materials and methods

### 2.1 Bioinformatics analysis

Our study utilized the TCGA-BRCA dataset to assemble a rigorously selected cohort comprising 1083 breast cancer cases, each with comprehensive clinicopathological profiles and high-quality whole-exome/transcriptome sequencing data. The analysis comprised only hormone receptor-positive breast cancer cases, totaling 676 cases (TCGA breast cancer cohort, TCGA BC cohort). We utilized the 21-gene signature as described in our previous study to classify the TCGA BC cohort into MDSCs^high^ and MDSCs^low^ groups [30]. Differential gene expression between these groups was assessed using the DESeq R software (1.8.3). Additionally, the biological processes and signaling pathways influenced by MDSCs^high^ were explored using the bioinformatics web portal (http://www.bioinformatics.com.cn/) to better interpret these findings.

### 2.2 Patient information

Shanghai Xinchao provided a tissue microarray from which 129 primary tissue samples from breast cancer were obtained (Table S1, Supporting Information). The surgical excision of these samples was place from January 2001 to August 2004. Only 67 cases of breast cancer that tested positive for hormone receptors were used for subsequent analysis. This study was conducted with approval from the Institutional Ethics Committee of Tianjin Medical University Cancer Institute and Hospital (Approval No. Ek2023111).

### 2.3 Cell culture

The E0771 cell (ATCC, CRL-3461) was sourced from the American Type Culture Collection and maintained in DMEM media, supplemented with 10 % fetal bovine serum. Cultures were incubated at 37 °C in a 5 % CO_2_ atmosphere. PIK3CA^WT^ and PIK3CA^E545K^ were established using the CRISPR/Cas9 system as previously described [14]. The cells were authenticated by short tandem repeat (STR) genotyping and routine mycoplasma testing to ensure the absence of contamination throughout the study.

### 2.4 Western blot

Protein extracts prepared with RIPA buffer were quantified by BCA assay. After denaturation, proteins were subjected to SDS-PAGE for electrophoretic separation and subsequently transferred onto PVDF membranes. To block the membranes, a 5 % non-fat milk solution was applied. Membranes were incubated sequentially with primary antibodies and HRP-conjugated secondary antibodies (Table S2, Supporting Information). Chemiluminescence detection was used to analyze the target proteins.

### 2.5 Cell isolation

To obtain MDSCs, GM-CSF (40 ng/mL) and IL-6 (20 ng/mL, both from PeproTech) were used to stimulate bone marrow cells for 72 h.

### 2.6 Flow cytometry

Monoclonal antibodies were used to stain single-cell suspensions, followed by gentle shaking to mix and incubation at room temperature for 20 min. The reaction was terminated with PBS, and after centrifugation, the cells were resuspended and sorted using a flow cytometer as described previously [31]. Complete antibody information is tabulated in Table S3 (Supporting Information).

### 2.7 Transwell assay

While the lower chamber of Transwell inserts held complete medium, the upper chamber was seeded with E0771 cells in serum-free medium. The Transwell chamber was pre-coated with or without 20 μL of Matrigel up to experimental requirements. Following 48h incubation, cells were fixed (4 % PFA), stained (0.1 % crystal violet), and gently rinsed with PBS. A cotton swab was used to remove any non-migrated cells from the top membrane, and PBS was then rinsed. An inverted microscope was used to view the transmigrated cells.

### 2.8 Quantitative real-time PCR

Using Trizol reagent, total RNA was extracted from cells or tissues. PrimerScript RT Master Mix was then used to convert the samples into cDNA (TaKaRa). Using the real-time PCR machine, quantitative real-time PCR was performed. The relative expression level of the gene was determined *via* the 2-ΔCt formula.

### 2.9 Cell transfection

Twenty-four hours before transfection, 400 μL of antibiotic-free media was used to seed cells in 24-well plates. Transfection was initiated at 30 %–50 % cell confluency (70 %–90 % for SIRT1 overexpression plasmid experiments). Transfections with SIRT1 overexpression plasmid, siRNAs targeting SIRT1 or Snail, miR-155-5p mimics/inhibitor, along with their respective controls were performed using Lipofectamine 2000. Cells were cultured for 48 h post-transfection, with a medium change 4–6 h after transfection.

### 2.10 Exosome purification and characterization

Cell cultures were maintained using fetal bovine serum from which exosomes had been removed. Supernatants were collected and subjected to differential centrifugation as described in the previous report [19]. Following the extraction, multi-modal assays were sequentially carried out on the exosomes, including particle-size analysis, electron-microscopy observation, and identification of protein markers, aiming to precisely define the relevant properties of the exosomes.

### 2.11 Dual-luciferase reporter assay

Using standard molecular cloning techniques, we inserted both wild-type and genetically modified 3′UTR regions of the SIRT1 gene into pGL3.0 plasmid backbones (Promega). For functional analysis, Lipofectamine 2000 reagent facilitated the simultaneous delivery of miR-155-5p synthetic oligonucleotides and the constructed reporter plasmids into cultured cells. Following a two-day incubation period, we quantitatively evaluated bioluminescence signals employing Promega's dual-reporter assay system to measure relative luciferase expression levels.

### 2.12 Animal experiment

Female C57BL/6J mice (6–8 weeks) were subcutaneously injected with of 5 × 10^5^ EO771^WT^/EO771^E545K^ cells. When xenografts reached >100 mm^3^, mice were randomized into treatment groups receiving alpelisib (20 mg/kg), cobomarsen (0.5 mg/kg), Mix (alpelisib + cobomarsen), alpelisib@MSPM, cobomarsen@MSPM, or cobomarsen@alpelisib-MSPM (5 mg/mL) *via* intraperitoneal injection every 2 days for six injections. Tumor volume (0.5 × length × width^2^) was measured every 3 days with simultaneous recording of body mass. Following a standardized protocol, euthanasia was performed under general anesthesia *via* cervical dislocation to harvest neoplastic tissues. These experimental protocols received formal approval from the Institutional Animal Care and Use Committee at Tianjin Medical University Cancer Institute and Hospital (Approval No. AE2023150).

### 2.13 Synthesis of PEG-b-PCL

The poly(ethylene oxide)-block-poly(ε-caprolactone) (PEG-*b*-PCL) was prepared through ε-caprolactone (ε-CL) ring-opening polymerization under controlled conditions. The reaction was conducted at 110 °C in toluene solvent, employing CH_3_O-PEG_114_-OH as the macroinitiator and stannous octoate (Sn(Oct)_2_) as the catalytic agent. The CH_3_O-PEG_114_-OH was subjected to vacuum drying for 24 h, and toluene and ε-CL were re-distilled to eliminate water. Briefly, CH_3_O-PEG_114_-OH (0.6 g, 0.12 mmol), ε-CL (1.14g, 0.01 mol), two drops of Sn(Oct)_2_ and 10 mL anhydrous toluene were added into a Schlenk tube. Following three freeze-pump-thaw cycles under argon, the reaction mixture was heated to 110 °C in an oil bath and maintained at this temperature for 12 h under an inert atmosphere. Then the solution precipitates in excess pre-cooled diethyl ether. The product was isolated by precipitation into an excess of chilled diethyl ether, followed by vacuum drying, yielding PEG_114_-*b*-PCL_70_ as a white solid.

### 2.14 Synthesis of PAE-b-PCL

The block copolymer poly(β-amino ester)-block-poly(ε-caprolactone) (PAE-*b*-PCL) was prepared through a combination of ring-opening polymerization of ε-caprolactone (ε-CL) and Michael addition polymerization. Initially, PCL-acrylate (PCL-A) was synthesized by dissolving ε-CL (2.71 g, 0.0238 mol), 2-hydroxyethyl acrylate (34.5 mg, 0.3 mmol), and two drops of Sn(Oct)_2_ in 10 mL of toluene, following the same procedure used for PEG-*b*-PCL synthesis. Then, PCL-A (0.5g, 0.057 mmol), Hexamethylene diacrylate (HDD) (0.196g,0.86 mmol) and 1,3-Di-4, piperidylpropane (TDP) (0.219g, 1.4 mmol) were dissolved in 10 mL of re-distilled CHCl_3_. The solution reacts at 55 °C for 72 h, then precipitates in excess precooled diethyl ether and is vacuum-dried to obtain white precipitate PCL-*b*-PAE.

### 2.15 Hydrophobicity of cobomarsen

The DOX·HCl aqueous solution (0.5 mL, 0.2 mg/mL) was gradually added into cobomarsen aqueous solution (0.5 mL, 5.0 nmol). Following a 10-min incubation at ambient temperature, the combined solution was subjected to centrifugation to separate the precipitate from the supernatant. The resulting sediment contained the hydrophobic cobomarsen complex [32].

### 2.16 Preparation and characterization of cobomarsen@alpelisib-MSPM

All polymeric micelles were prepared by the method of nano-coprecipitation. In brief, a mixture containing [cobomarsen&DOX] complex, PEG-*b*-PCL and PAE-*b*-PCL was dissolved in anhydrous THF (ratio 1:1, 5 mg/mL). Under ultrasonic conditions, the polymer solution was dropped into 7 mL acid water(pH∼5.0). The solution was transferred into a dialysis membrane with a molecular weight cut-off of 10 kDa and purified against PBS buffer for 24 h to eliminate residual THF. Then alpelisib (30 mg/mL) was added to cobomarsen@MSPM (0.5 mg/mL, 10 mL), ultrasonic for 20 min, the mixed system was transferred to a dialysis bag (MWCO = 5 kDa), dialysis in PBS solution for 2 h, centrifuged with an ultrafiltration tube (MWCO = 3 kDa), and then resuspended in PBS to obtain cobomarsen@alpelisib-MSPM. The final polymeric micelles were concentrated to 0.5 mg/mL. All remaining MSPM formulations were synthesized following the identical preparation protocol used for the cobomarsen@alpelisib-MSPM system.

### 2.17 Characterization of MSPMs

Approximately 2 mL aliquots of each sample were sterilized by filtration through 0.22 μm Millipore membranes into sterile scintillation vials. Dynamic light scattering (DLS) was used to characterize the hydrodynamic diameter distribution of the polymeric micelles at a 90° scattering angle. TEM characterization was conducted at 200 kV. For sample preparation, 10 μL aliquots were deposited onto carbon-coated copper grids and allowed to adhere for 10 min, then removing excess liquid with filter paper, and then negative staining with 2 % phosphotungstic acid for 5 min. The zeta potential of MSPMS in PBS at pH 5.0–7.4 was determined by ZETAPALS/BI-200SM.

### 2.18 Determining the stability of cobomarsen@alpelisib-MSPM

To systematically evaluate the colloidal stability of cobomarsen@alpelisib-MSPM, the micelles were separately introduced into PBS and PBS with 10 % FBS. The initial concentration of the micelles in all systems was standardized to 0.5 mg/mL. Both incubation systems were transferred to a 37 °C constant-temperature incubator and maintained under gentle shaking conditions. DLS was employed to monitor the particle size of cobomarsen@alpelisib-MSPM over a 7-day period. The protocols for DLS were consistent with those described in the preceding section; each sample was subjected to triplicate measurements, and the average values were adopted for analysis.

### 2.19 Encapsulation efficacy

The encapsulation efficiency (EE) of cobomarsen@alpelisib-MSPM was determined using an ultraviolet–visible spectrophotometer and a fluorescence spectrophotometer. Specifically, the micelles were completely disrupted by adding three volumes of THF, followed by the addition of heparin sodium (1 mg/mL) to dissociate the interaction between DOX and cobomarsen, thereby releasing free DOX. The concentration of alpelisib was quantified using ultraviolet spectroscopy, while DOX was quantified by fluorescence spectroscopy (Ex = 490 nm, Em = 590 nm). EE% = weight of the drug in micelles/weight of the total drug × 100.

### 2.20 Measurement of alpelisib release *in vitro*

The standard curve for alpelisib in PBS was first established by measuring the absorption values of alpelisib's PBS solution at 314 nm and generating fitting curves. The PBS solution was adjusted to pH 7.4 and pH 6.5 with hydrochloric acid solution (1 M) to simulate blood, normal tissue, and tumor microenvironment, respectively. Next, cobomarsen@alpelisib-MSPM was transferred to a dialysis tube (MWCO = 10 kDa). The system was maintained at 37 °C with continuous agitation at 100 rpm while immersed in 14 mL of buffer solution. At predetermined intervals, the released dialysate (1 mL) is removed and the same volume of fresh dialysate is replaced. The absorption values of dialysate at 314 nm were determined by UV–VIS spectroscopy.

### 2.21 Cell viability assay

A total of 7000 E0771 cells per well were seeded into 96-well plates and allowed to attach overnight. The next day, the medium was replaced with fresh culture medium supplemented with varying concentrations of MSPM, and the cells were incubated at 37 °C for 24 h. Following exposure, cells were rinsed with PBS and subsequently treated with 100 μL of CCK-8 reagent mixture (CCK-8 solution to DMEM ratio 1:9) for a 2-h incubation period. Absorbance was recorded at 450 nm, and cell viability was determined relative to the PBS-treated control group. To evaluate the tumoricidal effect of MSPM loaded with different components,10,000 E0771 cells were seeded into each well in 96-well plates and allowed to attach overnight. The next day, the medium was replaced with fresh culture medium (adjusted to pH 6.5 with 0.1 M hydrochloric acid) supplemented with DOX@MSPM, alpelisib@MSPM, cobomarsen@MSPM, and cobomarsen@alpelisib-MSPM (each incorporating DOX, alpelisib, or cobomarsen at identical concentrations to those present in cobomarsen@alpelisib-MSPM), and the cells were incubated at 37 °C for 24 h. Following exposure, cells were rinsed with PBS and subsequently treated with 100 μL of CCK-8 reagent mixture (CCK-8 solution to DMEM ratio 1:9) for a 1-h incubation period. Absorbance was recorded at 450 nm, and cell viability was determined relative to the PBS-treated control group.

### 2.22 Hematoxylin and eosin staining

Following euthanasia, major organs were harvested from the mice and they were preserved for 48 h in 4 % neutral buffered formalin. After being cut into 1 × 1 × 0.3 cm^3^ specimens, the tissues were dehydrated using a series of graded ethanols, cleaned in xylene, and embedded in paraffin. Tissue sections with a thickness of 10 μm were prepared using a rotary microtome. These slices were subsequently stained with H&E and seen under bright-field microscopy.

### 2.23 Multiplex immunohistochemistry

Multiplex immunohistochemistry staining followed our prior protocol [14]. Following deparaffinization, tissue sections were subjected to antigen retrieval using heated citrate buffer. Tissue sections were incubated first with primary antibodies at 4 °C overnight, then with HRP-linked Opal secondary antibodies, after blocking non-specific binding sites. Signal amplification was achieved using tyramide-based chemistry (Opal 4/7-Color Kits, AKOYA Biosciences) according to manufacturer protocols. Antibody stripping *via* microwave enabled sequential multiplex labeling. Sections were counterstained with DAPI and mounted with ProLong Gold (Invitrogen). A complete list of antibodies employed in this study is provided in Table S2 (Supporting Information).

### 2.24 Statistical methods

Statistical evaluations were conducted utilizing GraphPad Prism version 8 and SPSS software version 23.0. Categorical data are summarized as median values and analyzed using the non-parametric chi-square (χ^2^) test. Statistical significance was defined as p < 0.05, with precise p-values provided in the respective figure legends.

## 3 Results

### 3.1 MDSCs promoted the migration and invasion of HR + breast cancer cells

**Fig. 1:**
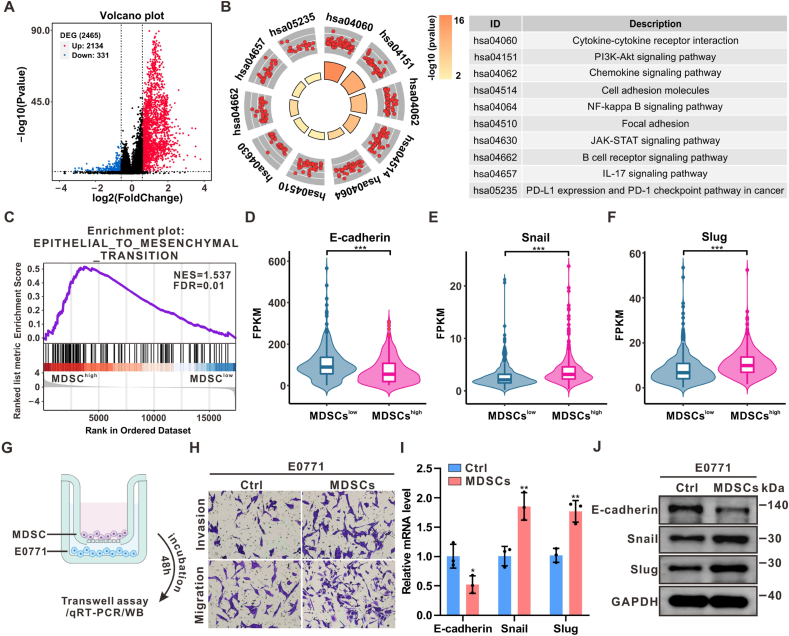
**MDSCs promoted migration and invasion of HR + breast cancer cells** (A-B) Volcano plot and KEGG analysis of significantly regulated genes in MDSCs^high^ within the TCGA BC cohort (n = 676). (C) GSEA enrichment plot for the epithelial-mesenchymal transition pathway in patients with high MDSCs infiltration in the TCGA BC cohort. (D–F) Distribution of FPKM values for three EMT-related genes in the TCGA BC cohort between MDSCs^high^ and MDSCs^low^ groups. (G) Schematic illustration of co-incubation of MDSCs and E0771 cells. (H) Transwell assays were conducted to assess migration and invasion of HR + breast cancer cells (E0771) after co-culturing with MDSCs. (I–J) The mRNA and protein abundances of E-cadherin, Snail, and Slug in E0771 cells were evaluated using qRT-PCR and Western blot following co-cultivation with MDSCs. n = 3. Data represents mean ± SD. ∗*P* < 0.05, ∗∗*P* < 0.01, ∗∗∗*P* < 0.001002E

In our previous research, a 21-gene expression signature was developed to quantitatively assess MDSC infiltration levels in breast tumor microenvironments [30]. Using this signature, HR + breast cancer patients from the TCGA breast cancer cohort (TCGA BC cohort) were classified into two groups: MDSCs^high^ and MDSCs^low^. Gene differential analysis was performed between these two groups. As shown in Fig. 1A, a total of 2465 genes were significantly regulated, with 2134 genes upregulated and 331 downregulated. KEGG enrichment analysis of the significantly regulated genes revealed substantial enrichment in cell adhesion molecules and focal adhesion, processes key to epithelial-mesenchymal transition (Fig. 1B) [33]. Additionally, GSEA enrichment analysis showed distinct upregulation of the EMT pathway in MDSCs^high^ patients (Fig. 1C). Given that E-cadherin suppression is a critical early event in EMT, the expression of E-cadherin and other EMT markers in the TCGA BC cohort was evaluated. The MDSCs^high^ group exhibited significantly reduced E-cadherin levels, alongside increased expression of Snail and Slug (Fig. 1D–F).

To investigate how MDSCs influence the metastatic potential of HR + breast cancer, bone marrow cells were extracted and induced from C57BL/6J mice, with CD11b + MDSC cells enriched by flow cytometry sorting for co-culture with HR + breast cancer E0771 cells (Fig. 1G). Transwell migration and invasion assays revealed a significant enhancement in the metastatic potential of E0771 cells following co-culture with MDSCs (Fig. 1H). Additionally, E-cadherin expression was significantly downregulated, while Snail and Slug were markedly increased in breast cancer cells after co-culturing with MDSCs (Fig. 1I–J). These results suggest that MDSCs enhance the migratory and invasive capacities of HR + breast cancer cells.

### 3.2 The MDSCs-derived exosomal miR-155-5p promoted migration and invasion of breast cancer cells

**Fig. 2:**
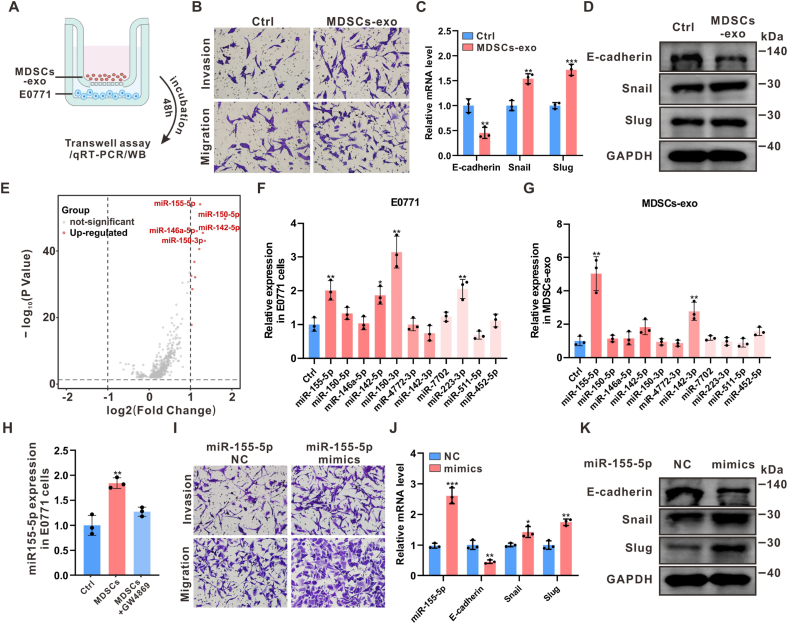
**The MDSCs-derived exosomal miR-155-5p promoted migration and invasion of breast cancer cells** (A) Schematic illustration of co-incubation of MDSC-derived exosomes (MDSCs-exo) and E0771 cells. (B) Transwell assays to evaluate migration and invasion capacity of E0771 cells after co-culturing with MDSC-derived exosomes. (C–D) The mRNA and protein levels of E-cadherin, Snail, and Slug in E0771 after co-culturing with MDSC-derived exosomes were assessed by qRT-PCR and Western blot. n = 3. (E) Volcano plot showing significantly regulated miRNAs in MDSCs^high^ from the TCGA BC cohort (n = 676). (F–G) The 11 significantly upregulated miRNAs in MDSCs^high^ breast cancer samples were determined by qRT-PCR in E0771 cells co-cultured with MDSC-derived exosomes. (H) Pre-treatment of MDSCs with GW4869 prior to co-culture significantly reduced miR-155-5p levels in E0771 cells. n = 3. (I) The miR-155-5p mimics (50 nM) significantly enhanced migration and invasion of E0771 cells. (J–K) qRT-PCR and Western blot was used to assess the mRNA and protein levels of miR-155-5p, E-cadherin, Snail, and Slug in E0771 cells after transfection of miR-155-5p mimics. n = 3. Data represents mean ± SD. ∗*P* < 0.05, ∗∗*P* < 0.01, ∗∗∗*P* < 0.001.

Exosome-mediated cell-cell communication is recognized as a key regulatory mechanism in TME remodeling [34,35]. To explore whether MDSC-derived exosomes enhance breast cancer cell motility and invasiveness, exosomes were purified from MDSC-conditioned medium (CM). The isolated exosomes were characterized by TEM for morphological analysis and nanoparticle tracking analysis for size distribution, confirming that the exosomes had diameters ranging from 50 to 150 nm (Fig. S1A–B, Supporting Information). Additionally, Western blot analysis revealed that exosome markers TSG101, Alix, and HSP70 were positive, while Calnexin, an endoplasmic reticulum marker absent in exosomes, was negative (Fig. S1C, Supporting Information), validating the purification of exosomes from MDSCs-CM. MDSC-derived exosomes also significantly enhanced the migratory and invasive behaviors of E0771 cells (Fig. 2A–B). Notably, significant changes in the expression of EMT markers E-cadherin, Snail, and Slug, both at the transcriptional and translational levels, were induced in E0771 cells by MDSC-derived exosomes (Fig. 2C–D). Furthermore, the exosomes facilitated the EMT process in a concentration-dependent manner (Fig. S1D–F, Supporting Information), mirroring the EMT phenotype seen in E0771 cells following direct co-culture with MDSCs.

To explore how MDSC-derived exosomes regulate the expression of EMT markers in tumor cells, we confirmed the presence of Snail transcripts in MDSC-derived exosomes by PCR (Fig. S1G, Supporting Information). However, after co-incubation with exosomes isolated from these Snail-knockdown MDSCs, the Snail mRNA and protein levels in the recipient cancer cells remained high and were not significantly reduced (Fig. S1H–I, Supporting Information). This finding strongly indicates that the MDSC-derived exosomes may be acting through the delivery of regulatory molecules that transactivate the endogenous Snail gene within the cancer cells.

miRNAs are critical molecular mediators that enable exosomes to regulate intercellular communication, selectively binding to post-transcriptional mRNAs and modulating gene expression either by degrading target mRNAs or suppressing protein production, thus influencing tumorigenesis and progression [36]. To determine if MDSC-derived exosomes promote breast cancer cell migration and invasion *via* a miRNA-dependent ceRNA network, differentially expressed miRNAs between MDSCs^high^ and MDSCs^low^ breast cancer samples were compared in the TCGA BC cohort. Eleven miRNAs were significantly upregulated in MDSCs^high^ breast cancer samples (Fig. 2E). This study further evaluated the expression of these 11 miRNAs in E0771 cells after co-incubation with MDSC-derived exosomes using qPCR and confirmed that miR-155-5p, miR-142-5p, miR-150-3p, and miR-223-3p were significantly upregulated (Fig. 2F). Among these, miR-155-5p exhibited the most significant upregulation in MDSC-derived exosomes (Fig. 2G). The treatment of MDSCs with a miR-155-5p inhibitor also led to a significant decrease in the level of miR-155-5p in the resulting exosomes (Fig. S1J, Supporting Information). Furthermore, the expression of miR-155-5p in E0771 cells markedly decreased when MDSCs were pre-treated with GW4869, an exosome secretion inhibitor, before co-culture (Fig. 2H). In contrast, GW4869 treatment did not significantly affect the expression of miR-155-5p within the MDSC-derived exosomes (Fig. S1J, Supporting Information), suggesting that MDSCs delivered miR-155-5p into tumor cells *via* MDSCs-derived exosomes.

Moreover, miR-155-5p was found to be aberrantly overexpressed in breast cancer tissues, correlating with poorer prognosis in the TCGA BC cohort (Fig. S2A–B, Supporting Information). Transfection with miR-155-5p mimics enhanced the migratory and invasive capacities of E0771 cells (Fig. 2I), while inducing an EMT phenotype characterized by E-cadherin downregulation and coordinated upregulation of Snail and Slug (Fig. 2J–K). Additionally, miR-155-5p inhibition effectively attenuated the pro-metastatic effects of MDSC co-culture on breast cancer cells and significantly modulated EMT marker expression (Fig. S2C–E, Supporting Information). These findings emphasize that MDSCs promote the migration and invasion of breast cancer cells *via* exosomal miR-155-5p.

### 3.3 miR-155-5p promoted the migration and invasion of breast cancer cells by directly inhibiting SIRT1

**Fig. 3:**
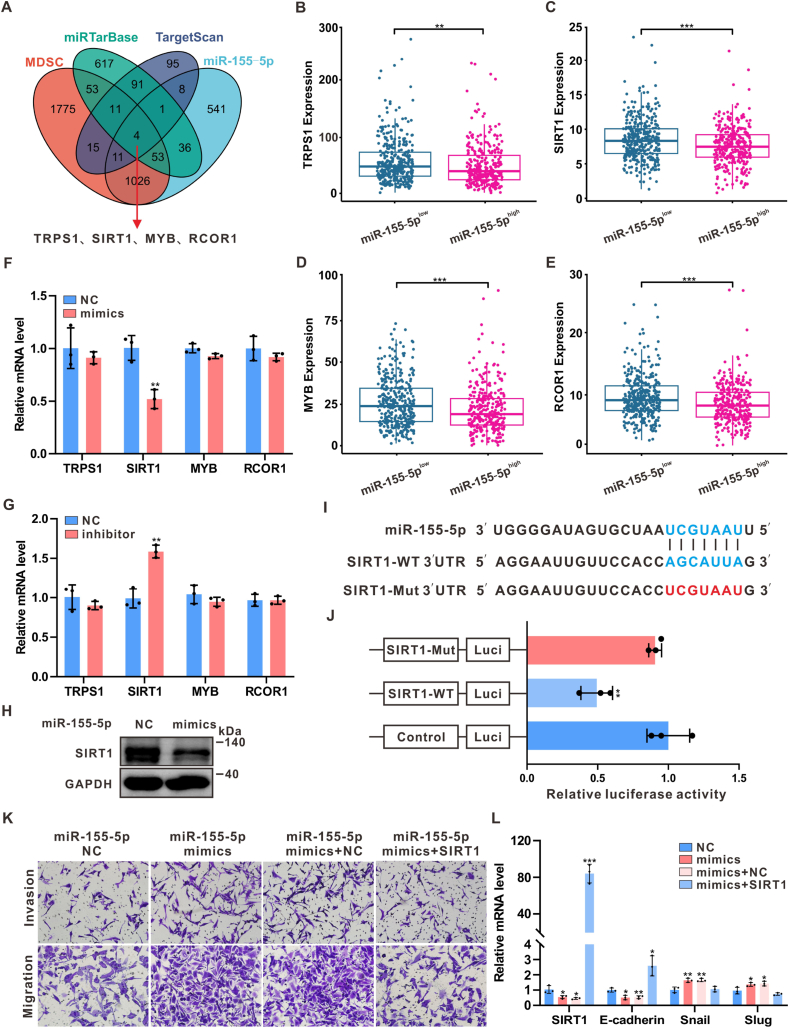
**miR-155-5p promoted migration and invasion of breast cancer cells by directly inhibiting SIRT1** (A) Venn diagram showing potential miR-155-5p target genes by TargetScan and miRTarBase. (B–E) TRPS1, SIRT1, MYB and RCOR1 exhibit marked downregulation in miR-155-5p^high^ group from the TCGA BC cohort (n = 676). (F–H) qPCR and Western blot confirmed that miR-155-5p mimics and inhibitor significantly regulate the expression level of SIRT1. n = 3. (I–J) Dual-luciferase assay verified direct binding of miR-155-5p to the 3′-UTR of SIRT1. n = 3. (K) Transwell assays were conducted to evaluate how SIRT1 overexpression modulates the metastatic potential of miR-155-5p mimic-transfected E0771 cells. (L) The mRNA changes of EMT-related markers after SIRT1 overexpression in E0771 cells transfected with miR-155-5p mimics were detected by qPCR. n = 3. Data represents mean ± SD. ∗*P* < 0.05, ∗∗*P* < 0.01, ∗∗∗*P* < 0.001.

To further investigate how MDSC-derived miR-155-5p promotes breast cancer invasion and migration, differential gene expression analysis was conducted between MDSCs^high^ and MDSCs^low^ groups, as well as between miR-155-5p^high^ and miR-155-5p^low^ groups in the TCGA BC cohort. Potential target genes of miR-155-5p were identified by Venn analysis using two bioinformatics tools, TargetScan and miRTarBase. This screening revealed four candidate genes: TRPS1, SIRT1, MYB, and RCOR1 (Fig. 3A). As shown in Fig. 3B–E, TRPS1, SIRT1, MYB, and RCOR1 were significantly downregulated in the miR-155-5p^high^ group. However, qPCR and Western blot experiments confirmed that miR-155-5p mimics and inhibitors significantly modulated SIRT1 expression levels exclusively (Fig. 3F–H). SIRT1, a protein deacetylase, plays a critical role in inhibiting the EMT process [37,38]. Knockdown of SIRT1 mimicked the pro-migratory and pro-invasive effects observed with miR-155-5p overexpression (Fig. S3A–B, Supporting Information), indicating that SIRT1 may be the direct downstream target of MDSCs-derived miR-155-5p in E0771 cells to enhance their aggressiveness.

Bioinformatic prediction analysis identified SIRT1 as a potential miR-155-5p target, with conserved binding sites in its 3′-UTR. To experimentally validate this interaction, luciferase reporter constructs containing different versions of the predicted binding motifs within the SIRT1 3′-UTR were generated (Fig. 3I). As demonstrated in Fig. 3J, luciferase activity was significantly suppressed in the presence of wild-type SIRT1 3′-UTR, while no effect was observed with mutant binding site controls, confirming direct targeting of SIRT1 by miR-155-5p. Furthermore, full-length SIRT1 overexpression counteracted miR-155-5p-induced migration, invasion, and EMT marker modulation in breast cancer cells (Fig. 3K–L). These data indicate that miR-155-5p enhances breast cancer cell aggressiveness by directly repressing SIRT1.

### 3.4 SIRT1 deficiency was detected in MDSCs^high^ HR + breast cancer tissues and promoted the EMT process

**Fig. 4:**
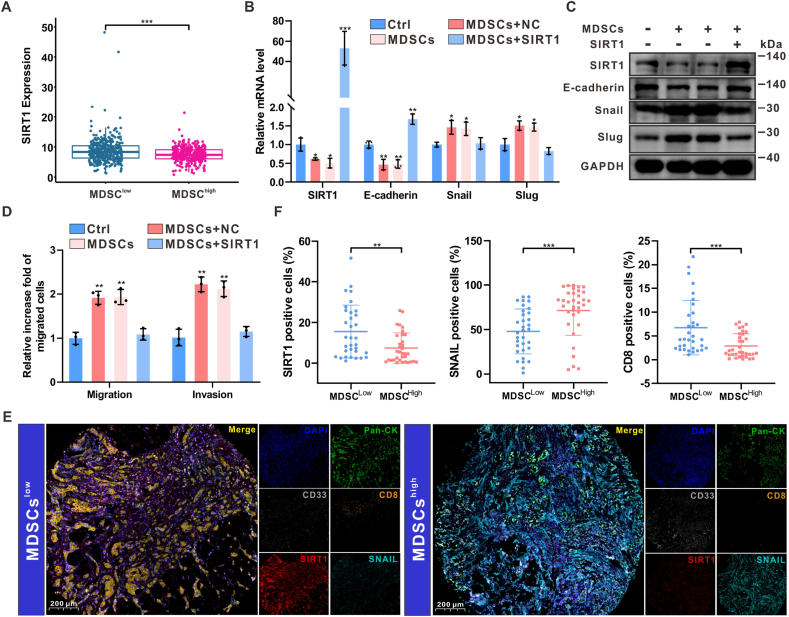
**SIRT1 deficiency was detected in MDSCs^high^ HR + breast cancer tissues and promoted the EMT process** **(A)** SIRT1 was downregulated in MDSCs^high^ breast cancer tissues of TCGA BC cohort (n = 676). (B–C) qPCR and Western blot confirmed the mRNA and protein levels of EMT-related markers after SIRT1 overexpression in E0771 cells co-cultured with MDSCs. n = 3. (D) Transwell assays to detect the effects of SIRT1-overexpression on migration and invasion of E0771 cells co-cultured with MDSCs. n = 3. (E–F) Representative mIHC images and quantification analysis showed the expression of SIRT1 and Snail in human breast tissue samples fron Xinchao BC cohort (n = 67), as well as T cell infiltration. Data represents mean ± SD. ∗*P* < 0.05, ∗∗*P* < 0.01, ∗∗∗*P* < 0.001.

As shown in Fig. S3C (Supporting Information), SIRT1 expression is significantly reduced in human breast cancer. Survival analysis of the TCGA BC cohort stratified by SIRT1 mRNA levels revealed that patients with low SIRT1 mRNA levels had shorter overall survival compared to those with high SIRT1 mRNA levels, highlighting the tumor suppressor role of SIRT1 in breast cancer (Fig. S3D, Supporting Information). Further investigation of MDSC-mediated regulation of SIRT1 expression in breast cancer tissues from the TCGA BC cohort revealed that SIRT1 was downregulated in the MDSCs^high^ group (Fig. 4A). Similarly, both SIRT1 mRNA and protein levels were significantly reduced in breast cancer cells co-cultured with MDSCs (Fig. 4B–C). Overexpression of SIRT1 reversed the EMT process as well as the migration and invasion abilities of breast cancer cells induced by MDSCs (Fig. 4B–D).

To further determine the relationship between MDSCs and SIRT1 expression in promoting the EMT process in HR + breast cancer, multiplex immunohistochemistry (mIHC) analysis was performed on an external validation cohort from Xinchao BC, which was divided into MDSCs^high^ and MDSCs^low^ groups based on MDSC infiltration levels. As shown in Fig. 4E–F, the MDSCs^high^ group exhibited low SIRT1 expression, high Snail expression, and low T cell infiltration. These results indicate that SIRT1 deficiency is present in MDSCs^high^ HR + breast cancer tissues, promoting the EMT process.

### 3.5 MDSCs promoted the growth and EMT of PIK3CA^MUT^ HR + breast cancer *via* activating the miR-155-5p/SIRT1 axis

**Fig. 5:**
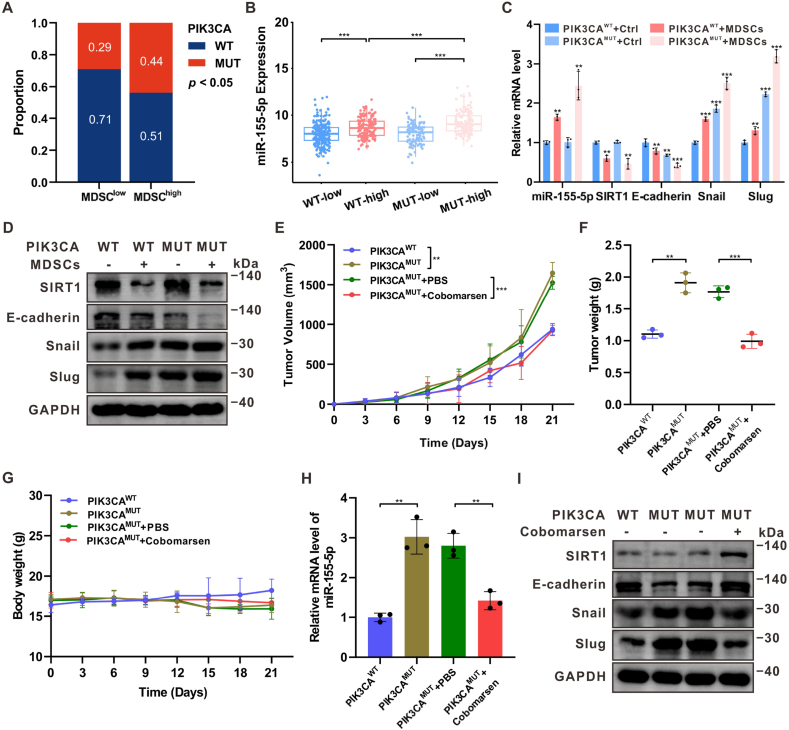
**MDSCs promoted the growth and EMT of PIK3CA^MUT^ HR** + **breast cancer *via* activating the miR-155-5p/SIRT1 axis** (A) In the TCGA BC cohort (n = 676), elevated MDSC infiltration showed a positive association with PIK3CA mutations. (B) High MDSC infiltration combined with PIK3CA^MUT^ revealed increased miR-155-5p expression in the TCGA BC cohort. (C) The mRNA levels of miR-155-5p, SIRT1 and EMT-related markers in PIK3CA^MUT^ HR + breast cancer cells after co-culturing with MDSCs. n = 3. (D) Western blot analysis of SIRT1 and EMT-related markers in PIK3CA^MUT^ HR + breast cancer cells after co-culturing with MDSCs. (E–G) General view of tumor volume, tumor weight, body weight from mice bearing PIK3CA^WT^ and PIK3CA^MUT^ E0771 xenografts (n = 3) treated with PBS and cobomarsen. (H–I) The mRNA level of miR-155-5p and the protein levels of SIRT1 and EMT-related markers in the above groups. Data represents mean ± SD. ∗∗*P* < 0.01, ∗∗∗*P* < 0.001.

PIK3CA mutations are commonly observed in breast cancer, with a particularly high prevalence in HR + subtypes. Analysis of MDSC infiltration in PIK3CA-mutated (PIK3CA^MUT^) breast cancer tissues from the TCGA BC cohort revealed significant MDSC accumulation in PIK3CA^MUT^ tumors (Fig. 5A). Further stratification of patient groups based on MDSCs^high^ and PIK3CA^MUT^ showed elevated expression levels of miR-155-5p (Fig. 5B). Consistent with these findings, significant correlations between miR-155-5p, SIRT1, and EMT markers were observed in PIK3CA^MUT^ HR + breast cancer cells following co-culture with MDSCs, suggesting activation of the miR-155-5p/SIRT1 axis in PIK3CA^MUT^ HR + breast cancer (Fig. 5C–D).

Previous studies have reported a high abundance of tumor-infiltrating MDSCs in PIK3CA^MUT^ E0771 xenografts [14]. To assess the impact of MDSCs on PIK3CA^MUT^ breast cancer progression, PIK3CA^WT^ or PIK3CA^MUT^ E0771 cells were subcutaneously implanted into C57BL/6J mice. After establishing xenografts, the mice were treated with either PBS or cobomarsen, an inhibitor of miR-155-5p. After 21 days, PIK3CA mutation accelerated tumor growth, and cobomarsen treatment resulted in a significant reduction in the average volume of PIK3CA^MUT^ tumors (Fig. 5E). Similarly, tumors with PIK3CA mutations in the cobomarsen-treated group showed significantly lower average weight compared to the control cohort (Fig. 5F). The body weight curve indicated no significant side effects during cobomarsen treatment (Fig. 5G). qPCR and Western blot analysis confirmed that cobomarsen treatment significantly reduced miR-155-5p, Snail, and Slug expression while increasing SIRT1 and E-cadherin levels (Fig. 5H–I). These results demonstrate that MDSCs promote PIK3CA^MUT^ HR + breast cancer growth and EMT by activating the miR-155-5p/SIRT1 signaling axis.

### 3.6 TME-responsive polymeric micelle cobomarsen@alpelisib-MSPM was synthesized and characterized

**Fig. 6:**
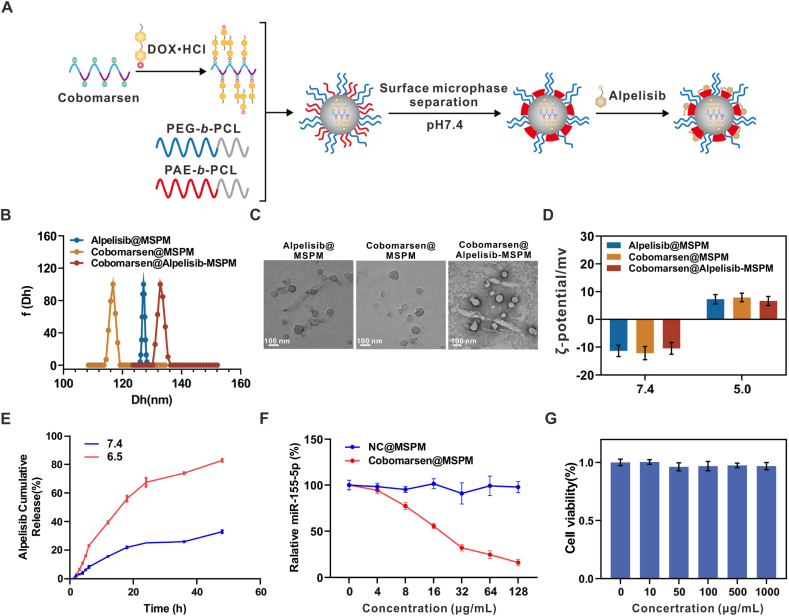
**TME-responsive polymeric micelles cobomarsen@alpelisib-MSPM was synthesized and characterized** (A) Schematic illustration for the preparation process for cobomarsen@alpelisib-MSPM. (B–D) Particle size distribution, TEM image and Zeta potential of alpelisib@MSPM, cobomarsen@MSPM and cobomarsen@alpelisib-MSPM. n = 3. Scale bar: 100 nm. (E) Cumulative release curve of alpelisib from alpelisib@MSPM at different pH values *in vitro*. n = 3. (F) The expression of miR-155-5p in PIK3CA^MUT^ E0771 cells after treated with different concentrations of cobomarsen@MSPM. n = 3. (G) The viability of E0771 cells following treatment with NC@MSPM. n = 3. Data represents mean ± SD.

Our previous work demonstrated that alpelisib, an effective anti-tumor agent, can reverses PIK3CA mutation-driven MDSCs recruitment [14]. Thus, we developed a pH-responsive amphiphilic block copolymer-based micellar system for the co-delivery of cobomarsen (a miR-155-5p inhibitor) and alpelisib (a selective PI3Kα inhibitor) to achieve TME-specific drug release and comprehensive MDSC inhibition in PIK3CA^MUT^ breast cancer. The system was constructed using synthesized PEG-*b*-PCL and PAE-*b*-PCL diblock copolymers (Fig. S4, Supporting Information), with successful polymerization confirmed by ^1^H NMR (Fig. S5, Supporting Information). The dual-loaded mixed-shell polymeric micelle (cobomarsen@alpelisib-MSPM) was prepared through a sequential assembly process (Fig. 6A). Briefly, cobomarsen was hydrophobized by complexation with DOX-HCl and subsequently incorporated into the micellar core with PEG-*b*-PCL and PAE-*b*-PCL in a weakly acidic solution. Increasing the pH to 7.4 triggered PAE deprotonation, causing it to collapse into hydrophobic microdomains on the PCL core, thus forming stable cobomarsen-loaded micelles. Alpelisib was then loaded through hydrophobic interactions with PAE, resulting in the final cobomarsen@alpelisib-MSPM formulation. Subsequent drug loading quantification revealed efficiencies of 62.15 % ± 1.0 % for alpelisib and 50.19 % ± 0.48 % for cobomarsen, confirming successful co-encapsulation. Characterization *via* DLS and TEM confirmed spherical nanostructures with uniform sizes (116–133 nm) (Fig. 6B–C). Furthermore, the micelles exhibited excellent colloidal stability, as evidenced by the highly consistent hydrodynamic diameter after 7-day incubation in PBS and 10 % FBS (Fig. S6, Supporting Information). Zeta potential measurements revealed pH-dependent surface charge reversal (Fig. 6D), validating the PAE chain's reversible phase transition and confirming the system's responsiveness to acidic TME conditions *in vivo*.

The choice of DOX·HCl was based on its well-documented ability to interact electrostatically with RNA, thereby conferring hydrophobic characteristics that facilitate efficient encapsulation into the hydrophobic core of the micelles [32]. To evaluate the contribution of doxorubicin in our nanoplatform, we compared the cytotoxicity of various MSPM formulations. As shown in Fig. S7, DOX@MSPM exhibited minimal cytotoxicity. Both alpelisib@MSPM and cobomarsen@MSPM (the complete formulation) showed significantly superior antitumor effects compared to DOX@MSPM, whereas cobomarsen@alpelisib-MSPM demonstrated markedly enhanced efficacy. These results clearly indicate that DOX doesn't exhibit a significant antitumor activity compared to the cobomarsen and alpelisib in cobomarsen@alpelisib-MSPM.

Next, the cumulative release behavior of alpelisib was assessed under varying *in vitro* pH conditions to evaluate the pH-responsive drug release profile. Notably, the release rate and cumulative amount of alpelisib significantly increased at pH 6.5, mimicking the acidic TME (Fig. 6E). This suggests preferential drug release within tumor tissues, thereby minimizing off-target effects in healthy tissues. The gene-silencing efficacy of cobomarsen@MSPM was also evaluated in PIK3CA^MUT^ E0771 cells. Quantitative analysis demonstrated that cobomarsen@MSPM significantly suppressed miR-155-5p in a concentration-dependent manner, while the negative control (NC@MSPM) showed no effect (Fig. 6F). Additionally, cytotoxicity assays revealed excellent biocompatibility of NC@MSPM across various concentrations, supporting the safety profile of the polymeric micelle system (Fig. 6G). These results substantiate that cobomarsen@alpelisib-MSPM functions as an efficient and biocompatible platform for the co-delivery of nucleic acids and small-molecule therapeutics.

### 3.7 Cobomarsen@alpelisib-MSPM suppressed tumor growth and reversed suppressive TME by inhibiting the miR-155-5p/SIRT1 axis in PIK3CA^MUT^ HR + breast cancer

**Fig. 7:**
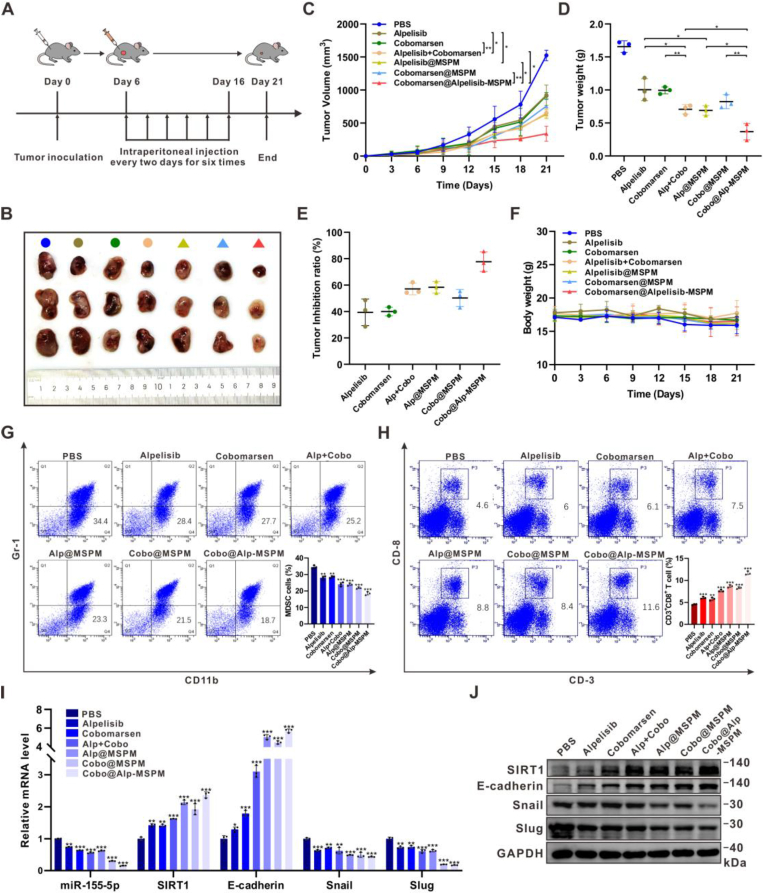
**Cobomarsen@alpelisib-MSPM suppressed tumor growth and reversed MDSCs-related TME remodeling by inhibiting the activation of miR-155-5p/SIRT1 axis in PIK3CA^MUT^ HR + breast cancer** (A) Schematic illustration of the therapy process *in vivo*. (B–F) General view of tumor mass, tumor volume, tumor weight, tumor inhibition ratio and body weight from mice bearing PIK3CA^MUT^ E0771 xenografts (n = 3) treated with PBS, alpelisib, cobomarsen, alpelisib + cobomarsen, alpelisib@MSPM, cobomarsen@MSPM and cobomarsen@alpelisib-MSPM. (G–H) Percentage of MDSCs and CD3^+^CD8^+^ T cells in the above groups. n = 3. (I–J) The mRNA and protein levels of miR-155-5p, SIRT1 and EMT-related markers in the above groups. n = 3. Data represents mean ± SD. ∗*P* < 0.05, ∗∗*P* < 0.01, ∗∗∗*P* < 0.001.

To assess the *in vivo* anti-tumor efficacy of the TME-responsive polymeric micelles cobomarsen@alpelisib-MSPM, xenograft models were established by subcutaneously implanting PIK3CA^MUT^ E0771 cells into female C57BL/6J mice (Fig. 7A). Seven treatment groups were randomly allocated: PBS (control), alpelisib, cobomarsen, alpelisib + cobomarsen, alpelisib@MSPM, cobomarsen@MSPM, and cobomarsen@alpelisib-MSPM. As shown in Fig. 7B–C, alpelisib@MSPM demonstrated superior anti-tumor effects compared to free alpelisib, likely due to enhanced drug accumulation in tumor tissues facilitated by micellar encapsulation. A similar trend, albeit less pronounced, was observed for cobomarsen@MSPM compared to free cobomarsen. While the combination of free drugs (alpelisib + cobomarsen) showed improved efficacy over single-agent treatments, the cobomarsen@alpelisib-MSPM co-delivery system achieved the most significant tumor growth suppression, highlighting the critical role of tumor-targeted delivery and enhanced cellular uptake in therapeutic efficacy. These results were further supported by tumor weight measurements and inhibition rate calculations (Fig. 7D–E). No significant changes were observed in body weight (Fig. 7F), and no histopathological abnormalities were detected in major organs (heart, liver, spleen, lungs, and kidneys), confirming the biocompatibility and safety of the micellar platform (Fig. S8, Supporting Information).

To evaluate the TME-remodeling capacity of the pH-responsive polymeric micelles, comprehensive immunological profiling was performed. Flow cytometry revealed a significant decrease in MDSCs and a concurrent increase in CD8^+^ T cells within tumors treated with alpelisib@MSPM and cobomarsen@MSPM compared to the corresponding free drug treatments (Fig. 7G–H), confirming enhanced tumoral accumulation and bioavailability due to micellar encapsulation. Notably, the immunomodulatory effects were further enhanced in the combination therapy group, with cobomarsen@alpelisib-MSPM showing the most pronounced reduction in MDSCs and increase in CD8^+^ T cells. This observation underscores the synergistic therapeutic effect arising from concurrent PI3K pathway inhibition and miR-155-5p blockade. Additionally, treatment with the micellar system significantly regulated the expression of miR-155-5p, SIRT1, and EMT markers at both the mRNA and protein levels (Fig. 7I–J). These results further validate the distinct anti-tumor efficacy of the TME-responsive polymeric micelle drug delivery system.

## 4 Discussion

In breast cancer, metastasis is responsible for the majority of fatalities, as the widespread distribution and aggressive nature of metastatic tumor cells diminish the effectiveness of cancer treatments [39,40]. The TME, a dynamically regulated network shaped by cell-to-cell interactions, plays a critical role in driving cancer progression and metastatic spread. Therefore, studying the crosstalk between cancer cells and their surrounding microenvironment is essential for understanding disease progression.

Exosomes serve as a key intercellular communication system in cancer biology, facilitating the transfer of molecular information between malignant cells and their surrounding environment to regulate tumor progression [[41], [42], [43]]. Our previous work demonstrated that tumor exosomal miR-9 (targeting SOCS3) and miR-181a (targeting PIAS3) synergistically activate the JAK/STAT signaling pathway, driving MDSC expansion and suggesting novel intervention strategies for IL-6-driven breast cancer [44]. Consistently, prostate cancer cells secreted PD-1 in exosomes that enhanced the activity of MDSC by activating JAK/STAT3 signaling [45]. Liu and colleagues showed that the transfer of GPR84 from MDSCs to CD8^+^ T cells *via* the exosomes attenuated the antitumor response [46]. In this study, we isolated MDSCs-derived exosomes from culture medium and investigated their functional role in breast cancer progression. *In vitro* co-culture experiments revealed that MDSC exosomes significantly enhanced the migratory and invasive capacities of breast cancer cells, indicating their critical role in metastatic dissemination.

The involvement of miRNAs in the development and progression of various cancer types is well-documented [47,48]. miR-155-5p has been identified as a differentially expressed miRNA in several cancers [49]. In breast cancer, Zhang et al. reported its upregulation and its role in promoting cell migration [50]. However, the expression profiles, functional contributions, and mechanistic roles of exosome-derived miR-155-5p in breast cancer progression remain insufficiently characterized. Our loss-of-function and gain-of-function experiments demonstrate that MDSCs promote the metastatic behavior of HR + breast cancer cells by transferring miR-155-5p *via* exosomes. Mechanistically, SIRT1 was identified as a direct functional target of miR-155-5p, which was validated by a luciferase reporter assay. The subsequent downregulation of SIRT1 is a pivotal event in the signaling cascade, establishing a direct mechanistic connection between MDSC-derived exosomal miR-155-5p and the acquisition of an enhanced invasive phenotype. These findings align with and expand the growing understanding of the pro-metastatic role of MDSCs within the tumor microenvironment [51,52]. The concept of bidirectional crosstalk is powerfully illustrated by the work of Shukla et al., who demonstrated that MDSCs and cancer cells engage in a mutualistic interaction that promotes the migration of both cell types [53]. This creates a self-reinforcing feedback loop that fuels tumor progression.

Leveraging this mechanism, a TME-responsive polymeric micelle co-delivering cobomarsen and alpelisib was developed, utilizing pH-sensitive release for spatiotemporal control and enhanced tumor-selective accumulation. A key challenge in dual-drug delivery lies in ensuring synchronized, targeted release of two pharmacologically distinct agents. Conventional methods often fail due to differences in drug properties (e.g., solubility, stability) and pharmacokinetics, leading to mismatched delivery ratios and off-target effects. pH-responsive polymeric micelles overcome these challenges by enabling co-encapsulation, ratiometric loading, and spatiotemporal control, enhancing therapeutic precision [54,55]. Acid-sensitive polymeric micelles, in particular, are highly effective in TMEs, where pH-triggered release increases drug accumulation and reduces premature degradation [[56], [57], [58]]. In this study, we successfully constructed a pH-sensitive amphiphilic block copolymer self-assembly polymer micelle and effectively loaded cobomarsen and alpelisib. The resulting cobomarsen@alpelisib-MSPM exhibited a uniform spherical structure. The PEG shell prolonged the systemic circulation duration of the polymeric micelles, while the protonation of PAE chains at low pH enabled the polymer micelles to respond to the acidic TME. Our results demonstrate that cobomarsen@alpelisib-MSPM can mediate the efficient delivery of cobomarsen and alpelisib to breast cancer cells. Additionally, the therapeutic efficacy of cobomarsen@alpelisib-MSPM was validated in a mouse model, where this dual-loaded polymeric micelle significantly inhibited tumor growth and reprogrammed the TME *in vivo*. Furthermore, targeting the PI3K signaling pathway suppressed tumor growth and significantly downregulated the miR-155-5p/SIRT1 axis within the tumors. This finding is contextualized by our prior research, which identified an upstream oncogenic driver of this pathway: PIK3CA mutations in breast cancer cells activate the PI3K/5-LOX/LTB4 axis to promote the recruitment and accumulation of MDSCs in the tumor microenvironment [14]. Consequently, we propose that PI3K inhibition exerts its effect, at least in part, by suppressing the upstream recruitment of MDSCs, which are critical for upregulating the miR-155-5p/SIRT1 pathway in tumor cells. Interestingly, we also observed that targeted inhibition of miR-155-5p/SIRT1 exhibited markedly reduced MDSC infiltration within the tumor microenvironment. This result aligns with our previous observation that miR-155-5p-mediated C/EBP-β downregulation triggers Wnt/mTOR signaling activation, which concurrently inhibits autophagy and induces a differentiation block in MDSCs [31]. These findings establish a novel TME-targeted therapeutic approach that disrupts metastatic cascades by coordinating microenvironmental and intracellular pathway modulation. However, it should be noted that our current study only evaluated the effects of the treatments on tumor growth and the EMT process *in vivo*, and lacks direct evidence regarding the subsequent step of metastasis. Further research is needed to systematically evaluate this process.

**Fig. 8:**
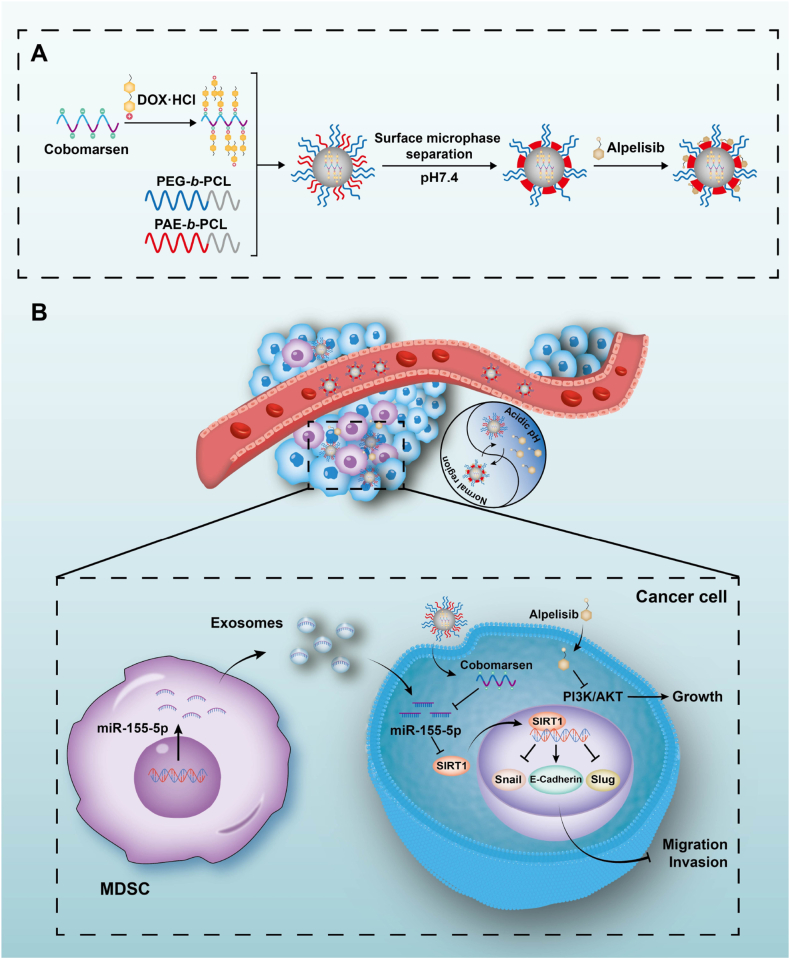
**Schematic illustration of synthetic procedures and therapeutic mechanisms of cobomarsen@alpelisib-MSPM** Exosomes secreted by MDSCs deliver miR-155-5p to HR + breast cancer cells, where it suppresses SIRT1 expression, accelerating tumor progression. To overcome this challenge, we developed a polymeric micelle targeting the tumor microenvironment to co-deliver cobomarsen and alpelisib, which demonstrated significant inhibition on PIK3CA-mutated HR + breast cancer growth and epithelial-mesenchymal transition.

To our knowledge, this study establishes that exosome-encapsulated miR-155-5p derived from MDSCs serves as both a critical pathogenic mediator and a promising therapeutic target in HR + breast cancer for the first time. As shown in Fig. 8, MDSCs-derived exosomal miR-155-5p promotes the migration and invasion of HR + breast cancer cells by targeting SIRT1. Furthermore, our findings highlight the therapeutic potential of the TME-responsive polymeric micelle cobomarsen@alpelisib-MSPM for breast cancer treatment, offering a novel approach to inhibit disease progression. Future preclinical investigations using refractory PIK3CA^MUT^ HR + breast cancer patient-derived organoids and xenograft-bearing mouse models should be conducted to further validate its therapeutic significance and clinical implications *in vitro* and *in vivo*.

## CRediT authorship contribution statement

**Guidong Chen:** Writing – original draft, Visualization, Formal analysis, Data curation, Conceptualization. **Silei Wang:** Writing – original draft, Visualization, Methodology, Investigation. **Fanchen Wang:** Investigation, Formal analysis, Data curation. **Chenju Yang:** Investigation, Data curation. **Rui Zhang:** Investigation. **Pengpeng Liu:** Methodology. **Junya Ning:** Formal analysis. **Shuyu Wang:** Software. **Feihe Ma:** Investigation. **Linlin Xu:** Writing – review & editing, Validation, Formal analysis, Conceptualization. **Linqi Shi:** Writing – review & editing, Project administration, Funding acquisition. **Jinpu Yu:** Writing – review & editing, Validation, Project administration, Funding acquisition.

## Declaration of competing interest

The authors declare that they have no known competing financial interests or personal relationships that could have appeared to influence the work reported in this paper.

## Data Availability

Data will be made available on request.
