# Supplementary material for: Targeted inhibition of MDSC-derived exosomal miR-155-5p restrains epithelial-mesenchymal transition in hormone receptor-positive breast cancer by regulating SIRT1
Source: Mater Today Bio. 2025 Oct 31;35:102492. doi: 10.1016/j.mtbio.2025.102492 (PMC12637072; doi:10.1016/j.mtbio.2025.102492)
Supplement: Multimedia component 1 [file mmc1.docx]

**Targeted inhibition of MDSC-derived exosomal miR-155-5p restrains epithelial-mesenchymal transition in hormone receptor-positive breast cancer by regulating SIRT1**

Guidong Chen^a,b,c,1^, Silei Wang^d,1^, Fanchen Wang^a,1^, Chenju Yang^a^, Rui Zhang^a,b,c^, Pengpeng Liu^a,b,c^, Junya Ning^a,b,c^, Shuyu Wang^a,b,c^, Feihe Ma^d^, Linlin Xu^d,🞰^, Linqi Shi^a,d,🞰🞰^, Jinpu Yu^a,b,c,🞰🞰🞰^

^a^Cancer Molecular Diagnostics Core, Tianjin Medical University Cancer Institute and Hospital, National Clinical Research Center for Cancer, State Key Laboratory of Druggability Evaluation and Systematic Translational Medicine, Tianjin’s Clinical Research Center for Cancer, Tianjin 300060, China

^b^Key Laboratory of Breast Cancer Prevention and Therapy, Tianjin Medical University, Ministry of Education, Tianjin 300060, China

^c^Key Laboratory of Cancer Immunology and Biotherapy, Tianjin 300060, China

^d^Key Laboratory of Functional Polymer Materials of Ministry of Education, State Key

Laboratory of Medicinal Chemical Biology, Institute of Polymer Chemistry, College of Chemistry, Nankai University, Tianjin 300071, China

^🞰^Corresponding author.

^🞰🞰^Corresponding author.

^🞰🞰🞰^Corresponding author.

*E-mail addresses:* jyu@tmu.edu.cn (J. Yu), shilinqi@nankai.edu.cn (L. Shi), llxu@mail.nankai.edu.cn (L. Xu).

^1^These authors contributed equally to this work.


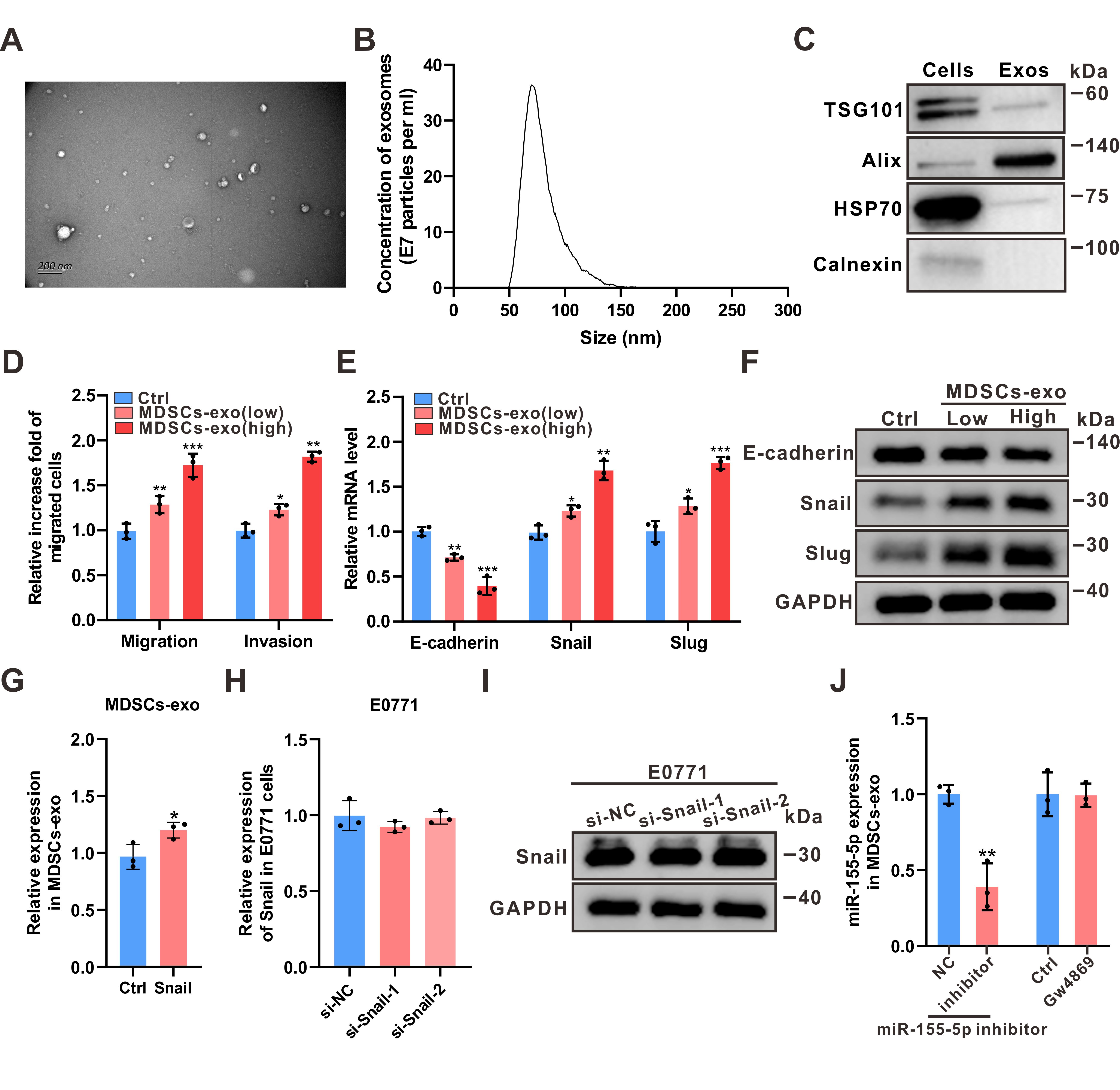


**Figure S1. MDSCs delivered miR-155-5p into breast cancer cells via exosomes to promote migration and invasion**

(A) Electron microscopic image of exosomes isolated from MDSCs. Scale bar: 200 nm. (B) The particle size distribution of MDSCs-derived exosomes was evaluated by Flow Nano Analyzer. (C) Western blot detected the indicated proteins in exosomes. (D) Transwell assays were conducted after HR+ breast cancer cells E0771 co-culturing with different concentrations of MDSC-derived exosomes. n = 3. (E-F) The mRNA and protein abundances of E-Cadherin, Snail, and Slug in E0771 were evaluated using qRT-PCR and Western blot following co-cultivation with different concentrations of MDSC-derived exosomes. n = 3. (G) The Snail transcripts were determined by qRT-PCR in MDSC-derived exosomes. n = 3. (H-I) The Snail mRNA and protein levels in the E0771 cells were determined by qRT-PCR and WB after co-incubation with exosomes isolated from these Snail-knockdown MDSCs. n = 3. (J) The miR-155-5p was determined by qRT-PCR in MDSC-derived exosomes after treated with miR-155-5p inhibitor and GW4869. n = 3. Data represents mean ± SD. ^🞰^P < 0.05, ^🞰🞰^P < 0.01, ^🞰🞰🞰^P < 0.001.

**
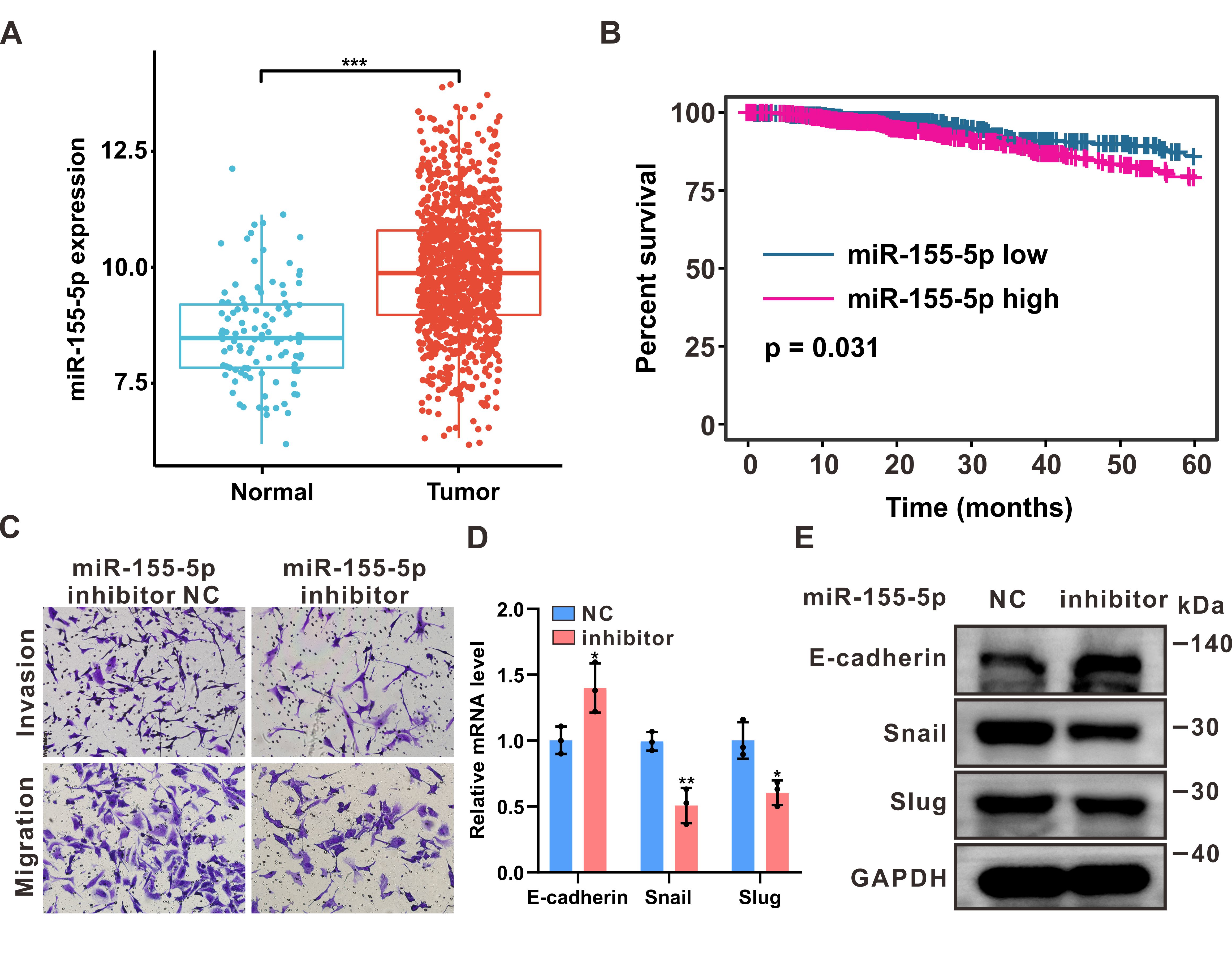
**

**Figure S2. miR-155-5p promoted migration and invasion of breast cancer cells**

(A) miR-155-5p was aberrantly overexpressed in breast cancer tissues compared to normal adjacent tissues (n = 1083). (B) miR-155-5p overexpression was significantly correlated with worse prognosis of breast cancer patients in TCGA BC cohort (n = 676). (C) Transwell assays to detect the migration and invasion ability of E0771 cells co-cultured with MDSCs in the presence or absence of the miR-155-5p inhibitor. (D-E) The mRNA and protein levels of EMT related markers in E0771 cells co-cultured with MDSCs in the presence or absence of the miR-155-5p inhibitor. n = 3. Data represents mean ± SD. ^🞰^*P* < 0.05, ^🞰🞰^*P* < 0.01, ^🞰🞰🞰^*P* < 0.001.

**
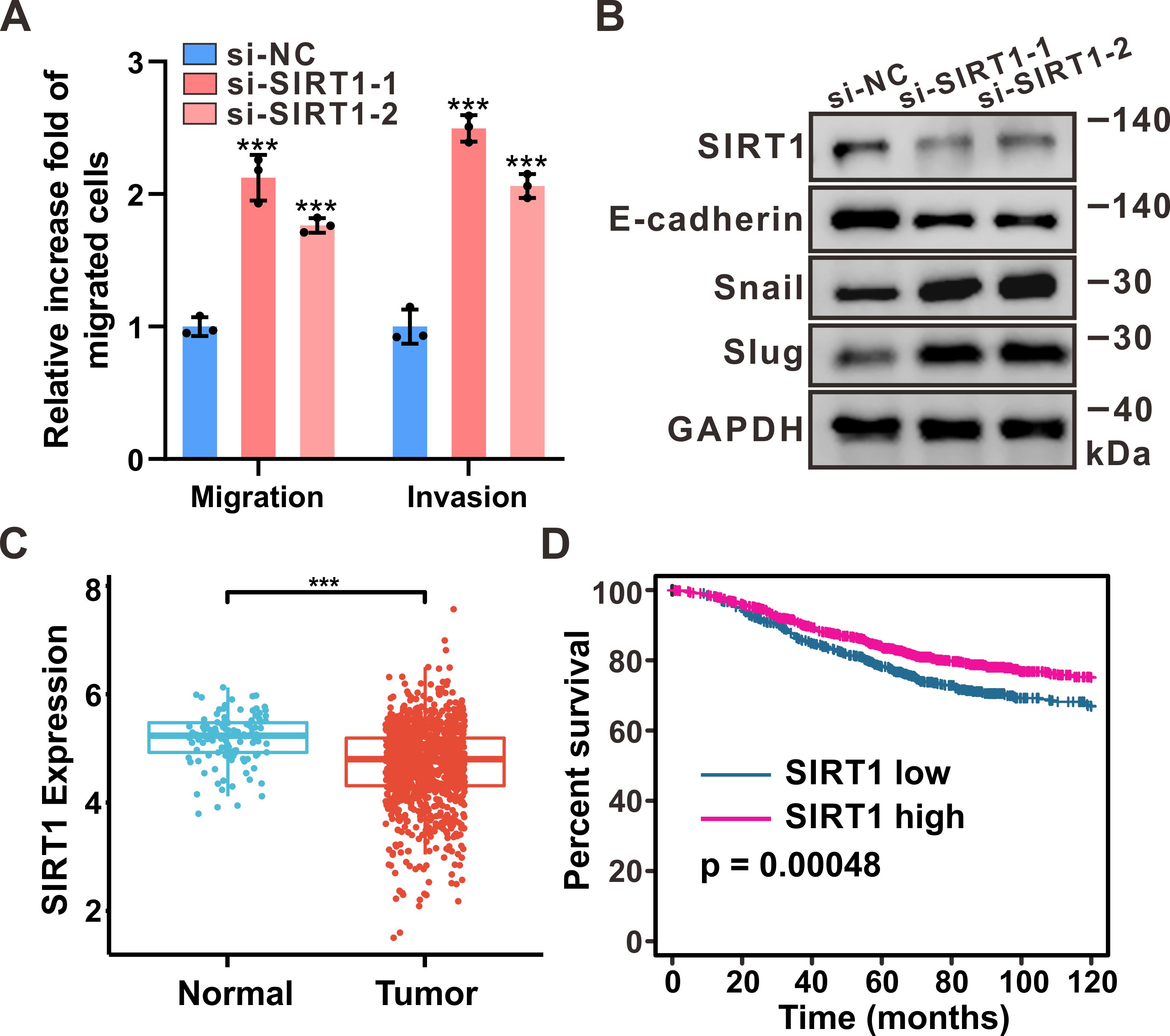
**

**Figure S3. SIRT1 leads to tumor progression by promoting the EMT process**

(A) Knockdown of SIRT1 significantly enhanced the migration and invasion ability of E0771 cells. n = 3. (B) Western blot was used to assess the protein levels of SIRT1, E-Cadherin, Snail, and Slug in E0771 cells after knockdown of SIRT1. (C) SIRT1 expression is significantly reduced in breast cancer tissues compared to normal adjacent tissues (n = 1083). (D) Low SIRT1 mRNA level was associated with shorter overall survival in TCGA BC cohort (n = 676). Data represents mean ± SD. ^🞰🞰🞰^*P* < 0.001.

**
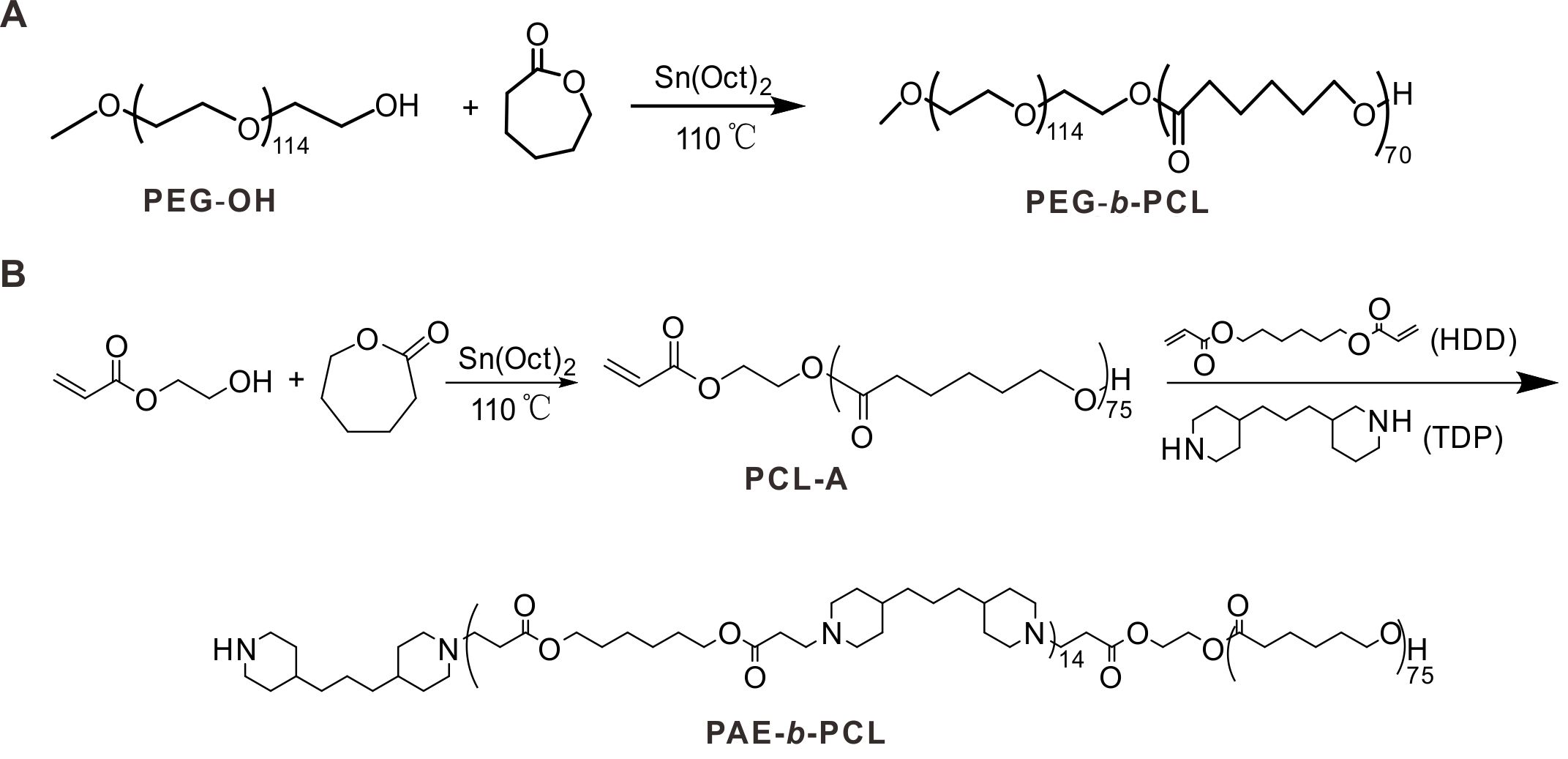
**

**Figure S4. Synthesis routes of (A) PEG-b-PCL and (B) PAE-*b*-PCL.**

**
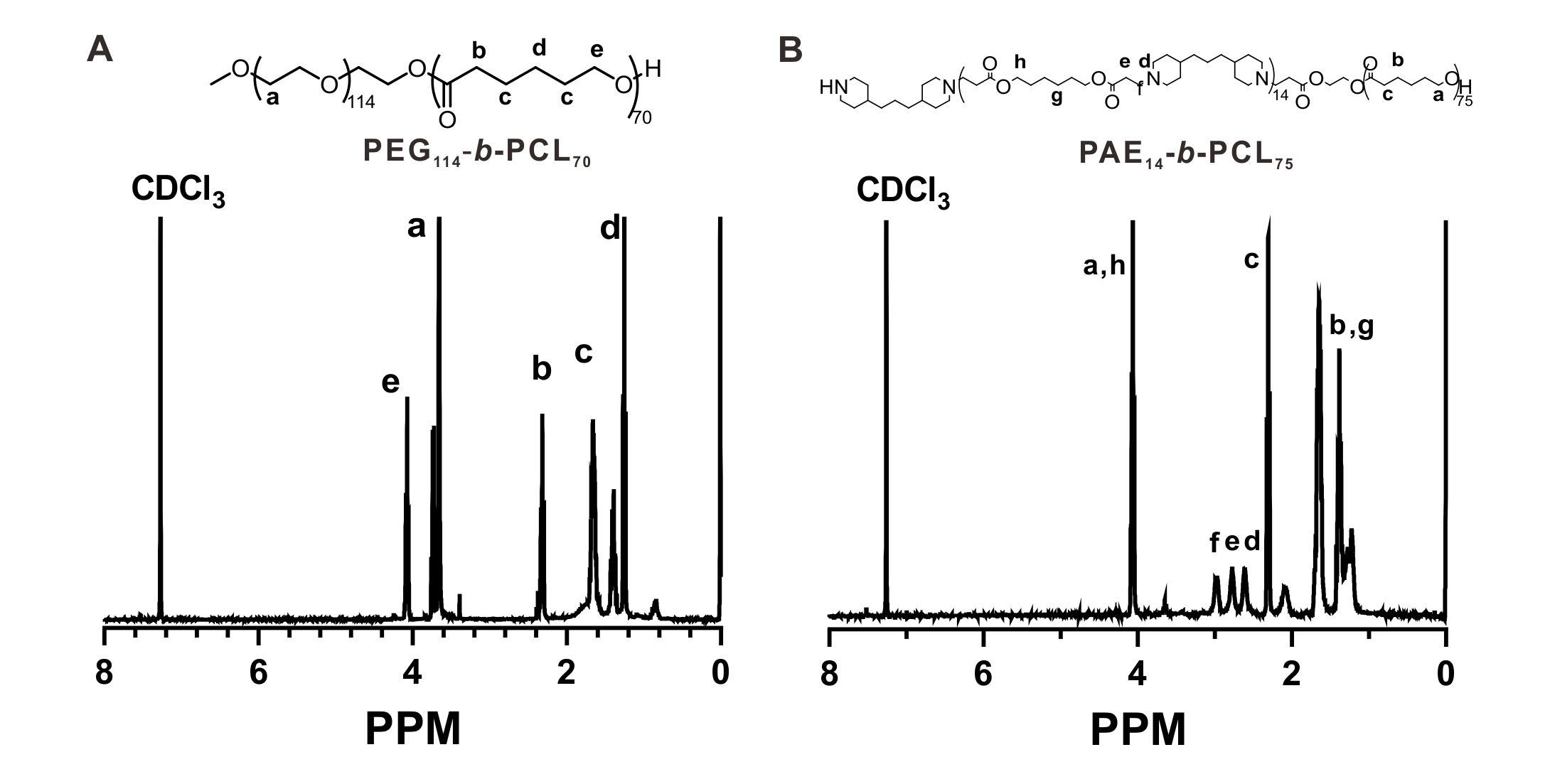
**

**Figure S5. ^1^H NMR spectrum of (A) PEG-b-PCL and (B) PAE-*b*-PCL.**


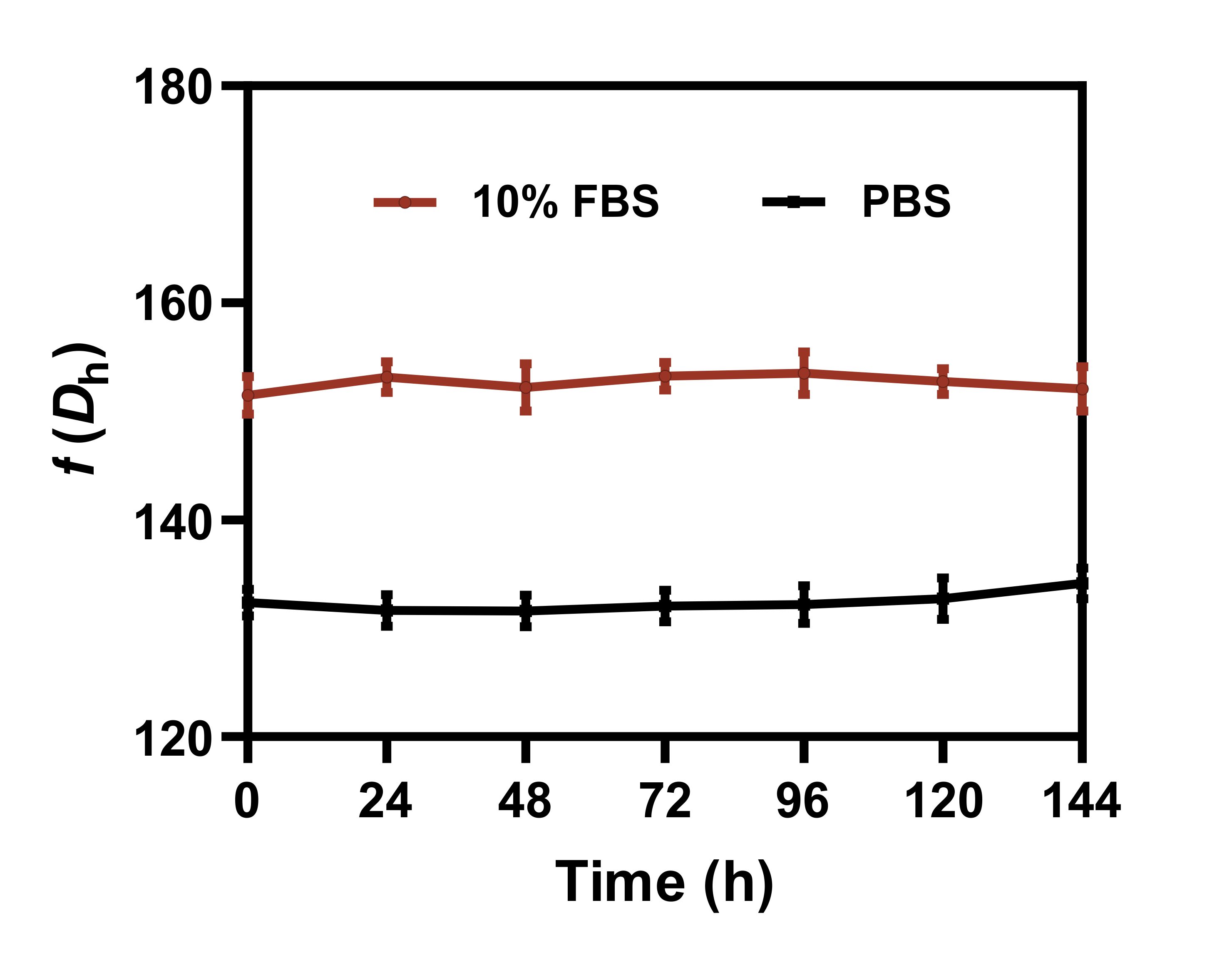


**Figure S6. Hydrodynamic diameter changes of polymeric micelles incubation in PBS and 10% FBS for 7 days. Data represents mean ± SD. n = 3.**


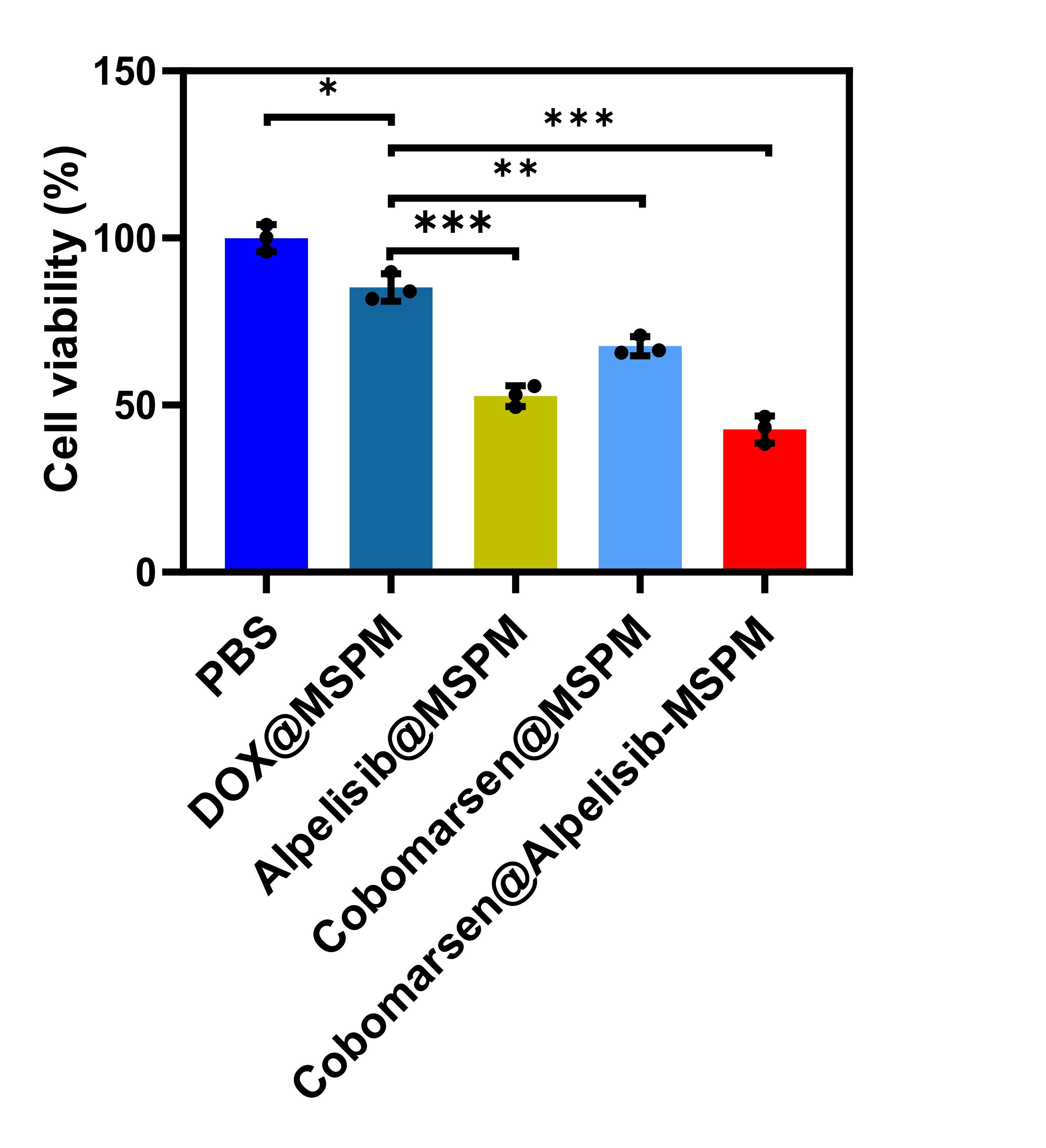


**Figure S7. Cytotoxicity of DOX@MSPM, alpelisib@MSPM, cobomarsen@MSPM and cobomarsen@alpelisib-MSPM against E0771 cells. n = 3. Data represents mean ± SD. ^🞰^*P* < 0.05, ^🞰🞰^*P* < 0.01, ^🞰🞰🞰^*P* < 0.001.**

**
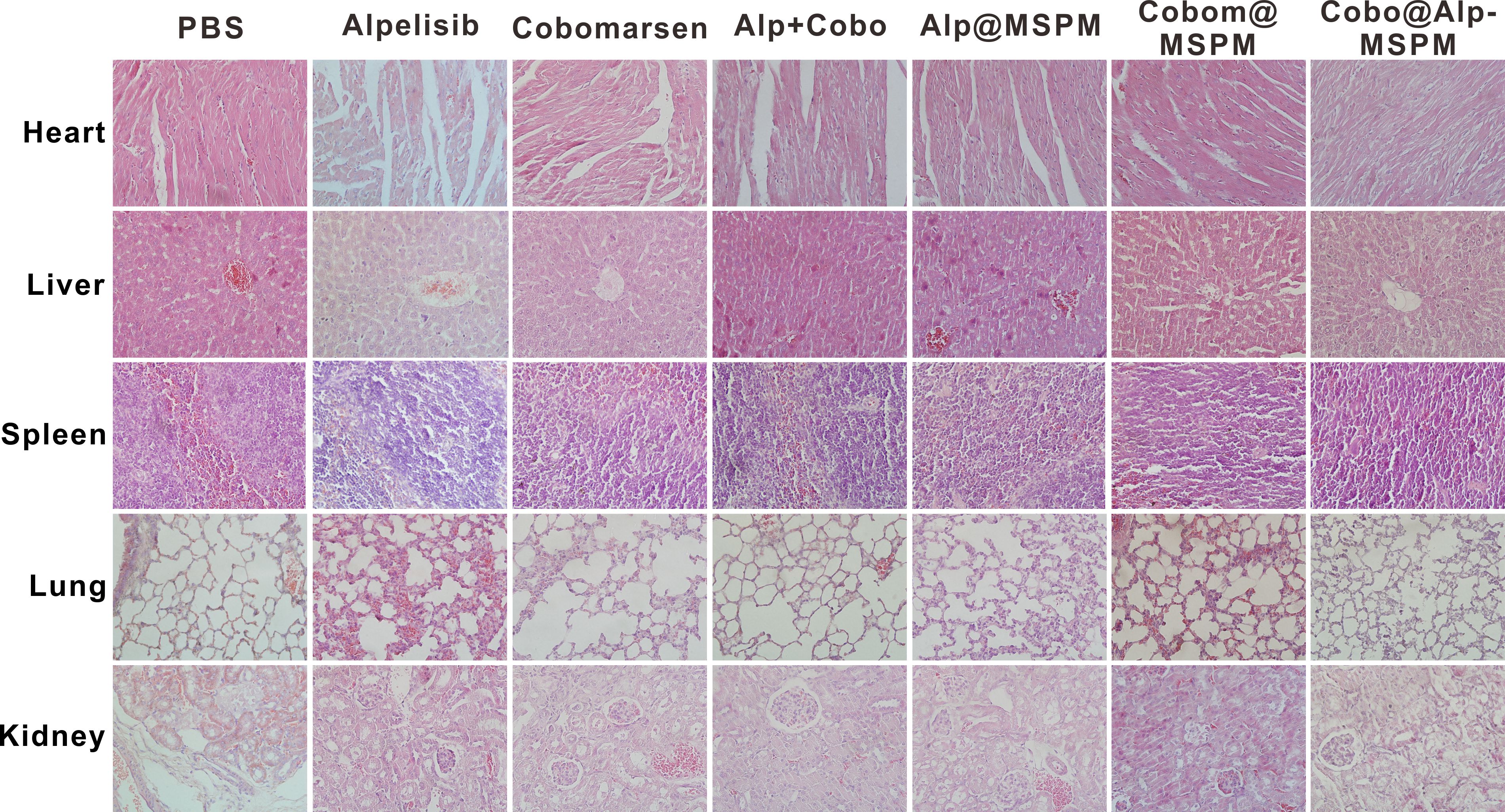
**

**Figure S8. H&E staining of the major organs from the mice after different treatments of PBS, alpelisib, cobomarsen, alpelisib+cobomarsen, alpelisib@MSPM, cobomarsen@MSPM and cobomarsen@alpelisib-MSPM.**

**Table S1.** Clinical pathology information about Xinchao BC cohort.

| **Clinical parameters** | **N/total** |
| --- | --- |
| Total | 129 |
| Age |  |
| ≤ 50 years | 59 |
| > 50 years | 70 |
| Tumor size |  |
| ≤ 2 cm | 22 |
| > 2 cm | 107 |
| Lymph nodes |  |
| Negative | 45 |
| Positive | 84 |
| Stage |  |
| I | 27 |
| II | 88 |
| III | 14 |
|  | |

**Table S2.** Key resources table.

| **Reagent/Resource** | **Dilution** | **Applications** | **Source** | **Identifier** |
| --- | --- | --- | --- | --- |
| **Antibodies** |  |  |  |  |
| E-Cadherin | 1:1000 | IB | Cell Signaling Technology | #3195 |
| Slug | 1:1000 | IB | Cell Signaling Technology | #80121 |
| Snail | 1:1000; 1:200 | IB; mIHC | Bioss | #bs-1371R |
| SIRT1 | 1:1000; 1:500 | IB; mIHC | Abcam | #ab189494 |
| GAPDH | 1:1000 | IB | Cell Signaling Technology | #2118 |
| CD33 | 1:500 | mIHC | Abcam | #ab269456 |
| Pan-CK | 1:250 | mIHC | Abcam | #ab7753 |
| CD8 | 1:5000 | mIHC | Proteintech | #66868-1-Ig |
| **Chemicals** |  |  |  |  |
| Alpelisib |  |  | MedChemExpress | #HY-15744 |
| Cobomarsen |  |  | TargetMol | #T88237 |
| IB: immunoblotting; mIHC: multiplex immunohistochemistry | | | | |

**Table S3.** Antibodies used in flow cytometry.

| **Antibodies** | **Company** | **Identifier** |
| --- | --- | --- |
| PE Rat Anti-Mouse CD8 | BD Biosciences | Cat#550798 |
| PerCP Rat Anti-Mouse CD45 | BD Biosciences | Cat#557235 |
| PerCP Rat Anti-Mouse CD3 | BD Biosciences | Cat#555274 |
| FITC Rat Anti-Mouse CD11b | BD Biosciences | Cat#557396 |
| PE Rat Anti-Mouse Gr-1 | BD Biosciences | Cat#553128 |
